# Morphology and secondary chemistry in species recognition of *Parmelia omphalodes* group – evidence from molecular data with notes on the ecological niche modelling and genetic variability of photobionts

**DOI:** 10.3897/mycokeys.61.38175

**Published:** 2019-12-11

**Authors:** Emilia Ossowska, Beata Guzow-Krzemińska, Marta Kolanowska, Katarzyna Szczepańska, Martin Kukwa

**Affiliations:** 1 Department of Plant Taxonomy and Nature Conservation, Faculty of Biology, University of Gdańsk, Wita Stwosza 59, PL-80-308, Gdańsk, Poland University of Gdańsk Gdańsk Poland; 2 Department of Geobotany and Plant Ecology, Faculty of Biology and Environmental Protection, University of Łódź, Banacha 12/16, 90-237, Łódź, Poland University of Łódź Łódź Poland; 3 Department of Biodiversity Research, Global Change Research Institute AS CR, Bělidla 4a, 603 00, Brno, Czech Republic Global Change Research Institute Brno Czech Republic; 4 Department of Botany and Plant Ecology, Wroclaw University of Environmental and Life Sciences, pl. Grunwaldzki 24a, PL-50–363, Wrocław, Poland Wrocław University of Environmental and Life Sciences Wrocław Poland

**Keywords:** Ascomycota, Parmeliaceae, parmelioid lichens, ITS rDNA, secondary metabolites, morphology, photobiont, ecological niche modelling

## Abstract

To evaluate the importance of morphological and chemical characters used in the recognition of species within the *Parmelia omphalodes* group, we performed phylogenetic, morphological and chemical analyses of 335 specimens, of which 34 were used for molecular analyses. Phylogenetic analyses, based on ITS rDNA sequences, show that *P. pinnatifida* is distinct from *P. omphalodes* and the most important difference between those species is the development of pseudocyphellae. In *P. pinnatifida*, they are mostly marginal and form white rims along lobes margins, but laminal pseudocyphellae can develop in older parts of thalli and are predominantly connected with marginal pseudocyphellae. In contrast, in *P. omphalodes* laminal pseudocyphellae are common and are predominantly not connected to marginal pseudocyphellae. Chemical composition of secondary lichen metabolites in both analysed species is identical and therefore this feature is not diagnostic in species recognition. Few samples of *P. discordans*, species morphologically similar to *P. omphalodes* and *P. pinnatifida*, were also included in the analyses and they are nested within the clade of *P. omphalodes*, despite the different chemistry (protocetraric acid present versus salazinic acid in *P. omphalodes*). All taxa of the *P. omphalodes* group occupy similar niches, but their potential distributions are wider than those currently known. The absence of specimens in some localities may be limited by the photobiont availability. *Parmelia omphalodes* and *P. pinnatifida* are moderately selective in photobiont choice as they form associations with at least two or three lineages of *Trebouxia* clade S. *Parmelia pinnatifida*, as well as *P. discordans* are associated with *Trebouxia* OTU S02 which seems to have a broad ecological amplitude. Other lineages of *Trebouxia* seem to be rarer, especially *Trebouxia* sp. OTU S04, which is sometimes present in *P. pinnatifida*. This study indicates the importance of extensive research including morphology, chemistry and analysis of molecular markers of both bionts in taxonomical studies of lichens.

## Introduction

The genus *Parmelia* Ach. (Parmeliaceae, Ascomycota) currently comprises ca. 40 species (Crespo and Lumbsch 2010; Thell et al. 2012; Molina et al. 2017) and was divided, based on the presence and type of vegetative diaspores, into three groups: the *P. saxatilis* group with isidiate species, the *P. sulcata* group containing sorediate species and the *P. omphalodes* group without vegetative propagules (Thell et al. 2017). To date, research has focused mainly on the isidiate and sorediate species (e.g. Molina et al. 2004, 2011, 2017; Divakar et al. 2005; Thell et al. 2008; Ossowska et al. 2018; Corsie et al. 2019; Haugan and Timdal 2019). The phylogenetic position of species of the *P. omphalodes* group and their taxonomic status have not been fully understood and required more detailed study as suggested by Molina et al. (2004) and Thell et al. (2008).

The *P. omphalodes* group includes three taxa, often treated at the species level, i.e. *P. discordans* Nyl., *P. omphalodes* (L.) Ach. and *P. pinnatifida* Kurok. (Hale 1987; Molina et al. 2004; Thell et al. 2008), but the distinction between them and their taxonomic status remain a long-term debate, especially in the case of *P. omphalodes* and *P. pinnatifida*. The first controversy concerns the taxonomic position of these species. Kurokawa (1976) presented the description of three species, *P. omphalodes*, *P. discordans* and *P. pinnatifida*, while Skult (1984) proposed a different concept and classified them as subspecies within *P. omphalodes*. Hale (1987) did not agree with Skult’s concept and distinguished two species within this group, i.e. *P. discordans* and *P. omphalodes*. He did not recognise *P. pinnatifida* as a separate species and included it in *P. omphalodes*.

The second issue is related to the differences between the species. Kurokawa (1976) noted that species of the *P. omphalodes* group differed in the shape of lobes and orientation of pseudocyphellae, which were mostly marginal in *P. pinnatifida*, whereas, in *P. discordans* and *P. omphalodes*, these were both laminal and marginal. In the case of the lobe shape, Kurokawa (1976) reported that *P. pinnatifida* has repeatedly branched lobes with narrow lobules, which are similar to those of *P. omphalodes*. *Parmelia discordans* has wider lobes than *P. pinnatifida* and without lobules, while *P. omphalodes* has the widest lobes with lobules. The descriptions in Skult (1984) indicated the same differences. The variation in lobe shape between *P. discordans* and *P. omphalodes* was also confirmed by Hale (1987), who classified both species in the group of taxa with marginal pseudocyphellae. Molina et al. (2004) and Thell et al. (2008) considered the shape of lobes and the orientation of pseudocyphellae as diagnostic features that distinguish both species; however, their conclusions were based mainly on published data, a limited number of specimens and few details about the species presented. In the discussion, they emphasised that those species required further studies. According to some works (e.g. Kurokawa 1976; Skult 1984; Hale 1987; Thell et al. 2008, 2011), differences in the secondary chemistry appear more diagnostic in the recognition of species within this group. Atranorin, salazinic and consalazinic acids, lobaric acid and protolichesterinic acid were reported as present in *P. omphalodes*. *Parmelia pinnatifida* is chemically similar, but lacks lobaric acid, whereas in *P. discordans* salazinic and consalazinic acids are replaced by protocetraric acid (e.g. Kurokawa 1976; Skult 1984; Thell et al. 2011).

The species of the *Parmelia omphalodes* group are rare in most parts of their distributional ranges. *Parmelia discordans* is reported from Europe only (Hale 1987; Hawksworth et al. 2008, 2011), whereas *P. omphalodes* and *P. pinnatifida* have wider geographical distributions and have been reported from Asia, Africa, Europe, South and North Americas (e.g. Hafellner 1995; Diederich and Sérusiaux 2000; Calvelo and Liberatore 2002; Hawksworth et al. 2008, 2011; Knežević and Mayrhofer 2009; Seaward 2010; Guttová et al. 2013; Esslinger 2015). Nevertheless, both those taxa are rarer than other members of the genus *Parmelia*. Furthermore, these species occupy similar habitats and grow mainly on siliceous rocks (Hale 1987; Thell et al. 2011).

According to literature, all *Parmelia* species form associations with green algae of the genus *Trebouxia* de Puymaly (Hale 1987; Friedl 1989; Nash 2008; Thell et al. 2011; Leavitt et al. 2015). Unfortunately, all studies to date focused mainly on species from *P. saxatilis* and *P. sulcata* groups and there are relatively fewer data on photobionts within the *P. omphalodes* group. Recent results showed that interactions between myco- and photobionts are not random, but depend on ecological or environmental factors, such as exposure or type of substratum, in addition to evolutionarily-determined specificity (Helms 2003; Peksa and Škaloud 2011; Leavitt et al. 2015). The prevailing view of symbiotic associations in lichens is that the mycobiont tends to form associations with photobionts best adapted to the local habitat conditions (Peksa and Škaloud 2011). Moreover, ecologically similar co-existing lichens may share the same pool of photobiont species (Rikkinen et al. 2002; Yahr et al. 2006). As species of *P. omphalodes* group grow mainly on rocks, one hypothesis, therefore, might be that the species should contain the same pool of *Trebouxia* species.

During our study of *P. omphalodes* and *P. pinnatifida* specimens, important differences between published data and the results of our own studies were observed. For example, lobaric acid was identified in the specimens with marginal pseudocyphellae (thus morphologically similar to *P. pinnatifida*) or both lobaric acid and fatty acids were absent in specimens with marginal and laminal pseudocyphellae (thus morphologically similar to *P. omphalodes*). The differences between our results and literature data prompted more detailed morphological, chemical and phylogenetic studies on those two species, which are also relatively common and thus easy to be sampled for molecular analyses. We also included a few samples of *P. discordans* to better understand the differences amongst all three species of *P. omphalodes* group, especially in the case of photobiont associations. In the study, we used the nuclear ribosomal internal transcribed spacer region (ITS), which is considered as a universal barcode marker for fungi in many taxonomic groups (e.g. Schoch et al. 2012; Leavitt et al. 2014; Divakar et al. 2016).

The main goals of this paper are to study the phylogenetic relationships between *P. discordans*, *P. omphalodes* and *P. pinnatifida*, to determine, based on molecular evidence, the diagnostic characters separating *P. omphalodes* and *P. pinnatifida* and to study the photobionts genetic variation in all three species. As not much is known about their ecology, the evaluation of the ‘ecological niche similarity’ is also presented.

## Materials and methods

### Taxon sampling

In total, 335 herbarium specimens deposited in B, H, HBG, LD, S, UGDA and UPS were used for morphological, chemical and ecological niche modelling (ENM) study: 61 of *P. discordans*, 113 of *P. pinnatifida* and 161 of *P. omphalodes*. A total of 34 specimens were selected for molecular study using the nuclear internal transcribed spacer region (ITS rDNA). Thirty four ITS rDNA sequences of the mycobionts and 17 ITS rDNA sequences of their photobionts were newly generated (Table 1). Additionally, 22 sequences from 10 *Parmelia* taxa and 67 representative sequences of *Trebouxia* OTUs, as proposed by Leavitt et al. (2015), were downloaded from GenBank. The specimens deposited in MAF herbarium, which sequences were also used here, have been morphologically and chemically analysed. Newly obtained ITS rDNA sequences were subjected to BLAST search (Altschul et al. 1997) in order to check their identity. All sequences have been deposited in GenBank (see Table 1).

**Table 1.** Specimens used in this study with the locality, voucher information, references and GenBank accession numbers. Sequences generated during this study are in bold.

Species/OTU	Voucher/ References	Fungal ITSrDNA	Algal ITSrDNA
*Parmelia discordans*	Sweden, S-F284965, Odelvik 15-293	**MN412798**	**MN412816**
	Sweden, S-F252494, Odelvik 13-147 et al.	**MN412800**	**MN412815**
	Sweden, UGDA L-23627, Kukwa 12278	**MN412799**	–
	UK, MAF-Lich 10232, (Molina et al. 2011)	AY583212	–
*Parmelia ernstiae*	Germany, HBG 4619 (Feuerer and Thell 2002)	AF410833	–
	Latvia, UGDA L-19917 (Ossowska et al. 2018)	KU845673	–
*Parmelia imbricaria*	Canada, TG 08-108 (Molina et al. 2017)	KT625503	–
*Parmelia mayi*	USA, MAF 15765 (Molina et al. 2011)	JN609439	–
	USA, MAF 15766 (Molina et al. 2011)	JN609438	–
	USA, MAF 15767 (Molina et al. 2011)	JN609437	–
*Parmelia omphalodes*	Sweden, S-F236118, Odelvik 12163	**MN412792**	**MN412806**
	Sweden, S-F300480, Odelvik 16-490	**MN412794**	**MN412805**
	Sweden, S-F252845, Odelvik 13-113	**MN412793**	**MN412808**
	UK, 2240 (Thell et al. 2008)	EF611295	–
	Finland (Thell et al. 2008)	AY251440	–
	Spain, MAF 7062 (Molina et al. 2004)	AY036998	–
	Spain, MAF 7044, (Molina et al. 2004)	AY036999	–
	Sweden, S-F238139, Odelvik 12238	**MN412796**	**MN412803**
	Sweden, UGDA L- 23632, Kukwa 12283	**MN412795**	**MN412817**
*Parmelia pinnatifida*	Norway, S-F254099, Odelvik 13-439	**MN412790**	**MN412804**
	Sweden, S-F299936, Odelvik 16-276	**MN412791**	–
	Sweden, S-F252763, Odelvik 13-225 et al.	**MN412797**	**MN412807**
	Sweden, S-F285120, Odelvik 15-294 et al.	**MN412789**	**MN412802**
	Poland, UGDA L-24300, Ossowska 118 et al.	**MN412774**	–
	Poland, UGDA L-24301, Ossowska 119 et al.	**MN412775**	**MN412813**
	Poland, UGDA L-24302, Ossowska 120 et al.	**MN412776**	–
	Poland, UGDA L-24304, Ossowska 123 et al.	**MN412777**	–
	Poland, UGDA L-24305, Ossowska 124 et al.	**MN412778**	**MN412814**
	Poland, UGDA L-24306, Ossowska 127 et al.	**MN412779**	–
	Poland, UGDA L-24307, Ossowska 132 et al.	**MN412780**	–
	Poland, UGDA L-24308, Ossowska 133 et al.	**MN412781**	–
	Poland, UGDA L-24310, Ossowska 137 et al.	**MN412783**	–
	Poland, UGDA L-24311, Ossowska 138 et al.	**MN412782**	–
	Poland, UGDA L-24318, Ossowska 150 et al.	**MN412785**	**MN412812**
	Poland, UGDA L-24319, Ossowska 152 et al.	**MN412784**	**MN412818**
	Poland, UGDA L-24313, Ossowska 143 et al.	**MN412786**	–
	Poland, UGDA L-24312, Ossowska 139 et al.	**MN412787**	**MN412811**
	Poland, UGDA L-24316, Ossowska 147 et al.	**MN412788**	–
	Poland, UGDA L-24294, Szczepańska s.n.	**MN412772**	**MN412810**
	Poland, UGDA L-24293, Szczepańska 1040	**MN412770**	**MN412809**
	Poland, UGDA L-24296, Szczepańska 1049	**MN412767**	–
	Poland, UGDA L-24297, Szczepańska 1052	**MN412768**	–
	Poland, UGDA L-24298, Szczepańska 1080	**MN412769**	–
	Poland, UGDA L-24295, Szczepańska 1126	**MN412773**	–
	Poland, UGDA L-24299, Szczepańska 1135	**MN412771**	–
	Austria (Thell et al. 2008)	EF611300	–
	Russia, MAF 7272 (Molina et al. 2004)	AY036988	–
	Russia, MAF 7274 (Molina et al. 2004)	AY036987	–
*Parmelia saxatilis*	Czech Republic, UGDA L-21245 (Ossowska et al. 2018)	KU845667	–
	Sweden, S-F300671, Odelvik 16-669 & Hedenäs	**MN412801**	–
	Sweden, MAF 6882 (Crespo et al. 2002)	AF350028	–
*Parmelia serrana*	Poland, UGDA L-21210 (Ossowska et al. 2018)	KU845669	–
	Spain, MAF 9756 (Molina et al. 2004)	AY295109	–
*Parmelia skultii*	Canada, LD 795 (Thell et al. 2004)	AY251456	–
	Greenland, 311C (Thell et al. 2004)	FJ425881	–
*Parmelia submontana*	Poland, UGDA L-21213 (Ossowska et al. 2018)	KU845664	–
	Morocco, MAF 15440 (Molina et al. 2011)	JN609434	–
*Parmelia sulcata*	Ireland, MAF 15421 (Molina et al. 2011)	JN118597	–
OTU I01	USA, I01_RH_shus_usa_UT_saxi_544 (Leavitt et al. 2015)	–	KR913803
OTU I02	USA, I02_ME_subau_usa_MI_cort_4176 (Leavitt et al. 2015)	–	KR913865
OTU I03	Estonia, I03_MH_exata_estonia_unk_cort_4110 (Leavitt et al. 2015)	–	KR913991
OTU I04	Russia, I04_RH_chryC_russia_Orenb_saxi_6890 (Leavitt et al. 2015)	–	KR914011
OTU I05	USA, I05_PUN_rud_usa_OH_cort_3157 (Leavitt et al. 2015)	–	KR914027
OTU I06	Canada, I06_MH_infum_canada_BC_saxi_4834 (Leavitt et al. 2015)	–	KR914029
OTU I07	USA, I07_ME_elber_usa_MN_cort_5773 (Leavitt et al. 2015)	–	KR914035
OTU I08	China, I08_MH_subexata_china_richuan_cort_3649 (Leavitt et al. 2015)	–	KR914042
OTU I09	USA, I09_MH_halei_usa_NC_cort_4008 (Leavitt et al. 2015)	–	KR914044
OTU I10	Argentina, I10_MH_ushua_argentina_unk_saxi_6045 (Leavitt et al. 2015)	–	KR914047
OTU I11	Russia, I11_MH_oliva_russia_Prim_cort_6012 (Leavitt et al. 2015)	–	KR914050
OTU I12	Russia, I12_MH_oliva_russia_Prim_cort_5998 (Leavitt et al. 2015)	–	KR914053
OTU I13	USA, I13_PUN_cas_usa_OH_cort_3161 (Leavitt et al. 2015)	–	KR914054
OTU I14	Russia, I14_MH_oliva_russia_Prim_cort_5973 (Leavitt et al. 2015)	–	KR914055
OTU I15	Kenya, I15_PUN_rud_kenya_unk_cort_1195 (Leavitt et al. 2015)	–	KR914056
OTU S01	Canada, S01_LE_lupina_canada_BC_cort_FJ170511 (Altermann 2009)	–	FJ170511
OTU S02	UK, S02_CE_acul_ant_shetland_terr_GQ375315 (Ruprecht et al. 2012)	–	GQ375315
OTU S03	Canada, S03_LE_vulpina_canada_BC_cort_FJ170752 (Altermann 2009)	–	FJ170752
OTU S04	Canada, S04_MH_exula_canada_BC_cort_5194 (Leavitt et al. 2015)	–	KR914114
OTU S05	USA, S05_LE_vulpina_usa_CA_cort_FJ170727 (Altermann 2009)	–	FJ170727
OTU S06	USA, S06_MH_eltula_usa_CO_cort_4212 (Leavitt et al. 2015)	–	KR914169
OTU S07	USA, S07_MH_eltula_usa_WA_cort_4343 (Leavitt et al. 2015)	–	KR914185
OTU S08	Spain, S08_CE_acul_spain_unk_terr_GQ375345 (Ruprecht et al. 2012)	–	GQ375345
OTU S09	Turkey, S09_CE_acul_turkey_unk_terr_GQ375351 (Ruprecht et al. 2012)	–	GQ375351
OTU S10	S10_TRE_simplex_SAG101_80_cult_FJ626735 (del Campo et al. 2010)	–	FJ626735
OTU S11	S11_TRE_australis_SAG2250_cult_FJ626726 (del Campo et al. 2010)	–	FJ626726
OTU S12	USA, S12_CE_acul_usa_AK_terr_GU124701 (Seifried 2009)	–	GU124701
OTU S13	S13_TRE_brindabellae_SAG2206_FJ626727 (del Campo et al. 2010)	–	FJ626727
OTU G01	Canaries, G01_PMT_pse_CANAR_gome_cort_3730 (Leavitt et al. 2015)	–	KR913271
OTU G02	Canaries, G02_PMT_per_CANAR_gome_cort_3751 (Leavitt et al. 2015)	–	KR913285
OTU G03	G03_TRE_usneae_UTEX2235_cult_AJ249573 (Friedl et al. 2000)	–	AJ249573
OTU G04	Canaries, G04_PMT_per_CANAR_gome_cort_3746 (Leavitt et al. 2015)	–	KR913286
OTU G05	G05_TRE_galapagensis_UTEX2230_AJ249567 (Friedl et al. 2000)	–	AJ249567
OTU A01	USA, A01_LEC_garov_usa_ID_saxi_078 (Leavitt et al. 2015)	–	KR912351
OTU A02	USA, A02_LEC_garov_usa_ID_saxi_108 (Leavitt et al. 2015)	–	KR912568
OTU A03	Sweden, A03_ME_fulig_swe_Skane_cort_3935 (Leavitt et al. 2015)	–	KR912760
OTU A04	USA, A04_XA_chE2_usa_ID_terr_201 (Leavitt et al. 2015)	–	KR912832
OTU A05	Mexico, A05_ORO_bicolor_mexico_OAX_cort_4043 (Leavitt et al. 2015)	–	KR912913
OTU A06	USA, A06_XA_coE3_usa_CO_saxi_6618 (Leavitt et al. 2015)	–	KR912989
OTU A07	USA, A07_XA_chE2_usa_UT_terr_008 (Leavitt et al. 2015)	–	KR913034
OTU A08	USA, A08_RH_mela_usa_UT_saxi_614 (Leavitt et al. 2015)	–	KR913115
OTU A09	USA, A09_XA_coE3_usa_UT_saxi_064 (Leavitt et al. 2015)	–	KR913162
OTU A10	Canada, A10_XA_cuF1_canada_BC_saxi_1007 (Leavitt et al. 2015)	–	KR913184
OTU A11	USA, A11_XA_idBX_usa_WY_terr_787 (Leavitt et al. 2015)	–	KR913199
OTU A12	USA, A12_XA_chE3_usa_UT_terr_126 (Leavitt et al. 2015)	–	KR913203
OTU A13	UK, A13_LEC_disp_uk_unk_saxi_6407 (Leavitt et al. 2015)	–	KR913212
OTU A14	USA, A14_XA_maricopF2_usa_A2_saxi_6699 (Leavitt et al. 2015)	–	KR913215
OTU A15	A15_TRE_gigantea_UTEX2231_cult_AF242468 (Kroken and Taylor 2000)	–	AF242468
OTU A16	Canada, A16_XA_caB1_canada_BC_terr_901 (Leavitt et al. 2015)	–	KR913224
OTU A17	Peru, A17_ORO_unk_peru_unk_cort_1602 (Leavitt et al. 2015)	–	KR913235
OTU A18	USA, A18_LEC_garov_usa_UT_saxi_140 (Leavitt et al. 2015)	–	KR913237
OTU A19	Canaries, A19_PMT_per_CANAR_gome_cort_3742 (Leavitt et al. 2015)	–	KR913241
OTU A20	USA, A20_XA_meF2_usa_A2_saxi_147 (Leavitt et al. 2015)	–	KR913248
OTU A21	USA, A21_XA_caB3_usa_ID_terr_334 (Leavitt et al. 2015)	–	KR913250
OTU A22	USA, A22_XA_chE2_usa_UT_terr_007 (Leavitt et al. 2015)	–	KR913255
OTU A23	A23_TRE_showmanii_UTEX2234_cult_AF242470 (Kroken and Taylor 2000)	–	AF242470
OTU A24	USA, A24_ME_calif_usa_CA_cort_4088 (Leavitt et al. 2015)	–	KR913251
OTU A25	USA, A25_XA_mariF2_usa_A2_saxi_6698 (Leavitt et al. 2015)	–	KR913259
OTU A26	USA, A26_XA_coE3_usa_UT_saxi_073 (Leavitt et al. 2015)	–	KR913261
OTU A27	USA, A27_XA_chE3_usa_WY_terr_110 (Leavitt et al. 2015)	–	KR913264
OTU A28	Mexico, A28_XA_diA1_mex_PU_saxi_098 (Leavitt et al. 2015)	–	KR913265
OTU A29	Japan, A29_MO_predis_japan_Shinano_saxi_8597 (Leavitt et al. 2015)	–	KR913266
OTU A30	USA, A30_XA_cuE2_usa_UT_saxi_036 (Leavitt et al. 2015)	–	KR913267
OTU A31	USA, A31_XA_coE1_usa_UT_saxi_030 (Leavitt et al. 2015)	–	KR913268
OTU A32	USA, A32_XA_cuE1_usa_UT_saxi_075 (Leavitt et al. 2015)	–	KR913269
OTU A33	A33_TRE_decolorans_UTEXB781_cult_FJ626728 (del Campo et al. 2010)	–	FJ626728
OTU A34	USA, A34_XA_mariF2_usa_AZ_saxi_6702 (Leavitt et al. 2015)	–	KR913270

### Morphology

The upper surfaces of all specimens were examined to determine the type of pseudocyphellae orientation such as: only marginal, marginal with few laminal in older parts of thalli and marginal and laminal in young and older parts of thalli. Pseudocyphellae were analysed on the whole thalli surfaces. Moreover, the length (distance between points of lobe branching) and width (distance between two adjacent lobe edges at the point of their branching) of lobes were also measured. Based on morphology and chemistry (see below), the studied specimens were divided into groups, which are characterised in Table 2. From each group (see Table 2) the samples were selected for DNA analysis.

**Table 2.** Diagnostic morphological and chemical features in species from *Parmelia omphalodes* group analysed in this study with their classification after molecular research (ATR – atranorin, SAL – salazinic acid with consalazinic acid, LOB – lobaric acid, PRC – protocetraric acid, LICH – lichesternic acid, PRL – protolichesterinic acid).

**Chemistry**	**Orientation of pseudocyphellae**	**Lenght (L) and width (W) of lobes (mm)**	**Voucher of specimens used in molecular research**	**Classification after molecular research**
**ATR, SAL, LOB**	marginal	L 1.5–2; W 1	S-F299936	*Parmelia pinnatifida*
			S-F254099	
**ATR, SAL, LOB**	marginal, laminal in older lobes	L 2; W 2	UGDA L-24310	*Parmelia pinnatifida*
			S-F252763	
**ATR, SAL, LOB, LICH, PRL**	marginal	L 1–2; W 0.5–1.5	UGDA L-24295	*Parmelia pinnatifida*
			UGDA L-24311	
			UGDA L-24319	
			UGDA L-24294	
			UGDA L-24296	
			UGDA L-24298	
			UGDA L-24305	
			UGDA L-24306	
**ATR, SAL, LOB, LICH, PRL**	marginal, laminal in older lobes	L 1.5–2; W 1.5–2	UGDA L-24313	*Parmelia pinnatifida*
			UGDA L-24308	
			UGDA L-24293	
			UGDA L-24297	
**ATR, SAL, LOB, PRL**	marginal	L 0.5–2; W 0.5–1	UGDA L-24299	*Parmelia pinnatifida*
			UGDA L-24300	
			UGDA L-24307	
			UGDA L-24318	
**ATR, SAL**	marginal	L 1; W 1	UGDA L-24304	*Parmelia pinnatifida*
			MAF 7274	
**ATR, SAL**	marginal, laminal in older lobes	L 1.5 ,W 1	UGDA L-24312	*Parmelia pinnatifida*
**ATR, SAL, LICH, PRL**	marginal	L 2; W 1	UGDA L-24301	*Parmelia pinnatifida*
**ATR, SAL, PRL**	marginal	L 1.5–2; W 1.5–1	UGDA L-24302	*Parmelia pinnatifida*
			S-F285120	
**ATR, SAL, PRL**	marginal, laminal in older lobes	L 1.5; W 1	UGDA L-24316	*Parmelia pinnatifida*
**ATR, PRC, LOB**	marginal	L 3; W 1–2	S-F284965	*Parmelia discordans*
			S-F252494	
			MAF 10232	
**ATR, PRC**	marginal and laminal on young thalli	L 3; W 2	UGDA L-23627	*Parmelia discordans*
**ATR, SAL, LOB**	marginal, laminal	L 3–4; W 2–3	S-F300480	*Parmelia omphalodes*
			S-F252845	
			S-F238139	
			S-F236118	
			UGDA L-23632	
			MAF 7044	
**ATR, SAL**	marginal, laminal	L 2; W 1.5	MAF 7062	*Parmelia omphalodes*

### Chemistry

Secondary lichen compounds were identified using thin-layer chromatography (TLC) in solvents A and C (Orange et al. 2001). The presence or absence of fatty acids was checked on two types of TLC plates: glass and aluminium. In order to check the differences in the concentration of lobaric acid in different parts of thalli, samples from marginal and central parts of thalli were analysed using TLC.

### DNA extraction, PCR amplification and sequencing

Total genomic DNA was extracted using the Sherlock AX Kit (A&A Biotechnology, Poland) in accordance with the manufacturer’s protocol, with slight modifications described by Ossowska et al. (2018).

Fungal ITS rDNA was amplified using the primers ITS1F and ITS4A (White et al. 1990; Gardes and Bruns 1993), while algal ITS rDNA was amplified using the following primers: LR3, ITS4M, ITS1T, ITS4T and AL1500bf (Friedl and Rokitta 1997; Kroken and Taylor 2000; Helms et al. 2001; Guzow-Krzemińska 2006). Amplification was performed in a total volume of 25 μl containing 1.0 μl of 10 μM of each primer, 12.5 μl of Start-Warm HS-PCR Mix Polymerase (A&A Biotechnology, Poland), 1.0 μl of dimethyl sulphoxide (DMSO), 3.0 μl of template DNA (~10–100 ng) and water.

The amplifications were performed in an Eppendorf thermocycler and carried out using the following programme: for fungal ITS rDNA marker: initial denaturation at 94 °C for 3 min and 33 cycles of: 94 °C for 30 sec; annealing at 52 °C for 45 sec; extension at 72 °C for 1 min and final extension at 72 °C for 10 min. For green-algal ITS: initial denaturation at 94 °C for 3 min and 35 cycles of: 94 °C for 45 sec; annealing at 55 °C for 45 sec; extension at 72 °C for 90 sec and final extension at 72 °C for 7 min.

The PCR products were purified using Wizard SV Gel and PCR Clean Up System (Promega, US), according to the manufacturer’s instruction. The cleaned DNA was sequenced using Macrogen sequencing service (http://www.macrogen.com).

### Phylogenetic analyses

The newly generated mycobiont sequences, together with selected representatives of *Parmelia* spp., were automatically aligned in Seaview (Galtier et al. 1996; Gouy et al. 2010) using the algorithm MUSCLE (Edgar 2004), followed by manual correction and elimination of terminal ends. Then, selection of unambiguously aligned positions was performed using Gblocks 0.91b (Castresana 2000) employing less stringent conditions. The final alignment of mycobionts consisted of 58 ITS rDNA sequences and 444 characters. A sequence of *P. sulcata* (JN118597) was used as an outgroup.

The newly generated photobiont sequences, together with representative *Trebouxia* OTUs, downloaded from Dryad database (Dryad Digital Repository) (Leavitt et al. 2015) and described in Leavitt et al. (2015), were automatically aligned using MAFFT – Multiple Alignment using Fast Fourier Transform (Katoh et al. 2002), as implemented in UGENE (Okonechnikov et al. 2012). It was followed with a selection of unambiguously aligned positions using Gblocks 0.91b (Castresana 2000) with less stringent settings (i.e. allowing smaller final blocks, gap positions within the final blocks and less strict flanking positions).

The final alignment of photobionts consisted of 84 ITS rDNA sequences and 580 characters. The names of operational taxonomic units (OTU) for *Trebouxia*ITS rDNA sequences were given according to Leavitt et al. (2015).

The GTR+I+G best-fit evolutionary model was selected for the mycobiont dataset, based on Akaike Information Criterion (AIC) (Akaike 1973) as implemented in MrModelTest2 (Nylander 2004). For photobionts, we used Partition Finder 2 (Lanfear et al. 2016), implemented at CIPRES Science Gateway (Miller et al. 2010) to determine the best substitution model for each partition under Akaike Information Criterion (AIC) and greedy search algorithm (Lanfear et al. 2012). Two different models were found for partitions, i.e. TRNEF+I+G for 5.8S and GTR+I+G+X for both ITS regions.

Bayesian analysis was carried out using the Metropolis-coupled Markov chain Monte Carlo (MCMCMC) method by using the Markov chain Monte Carlo (MCMC) method, in MrBayes v. 3.2.6 (Huelsenbeck and Ronquist 2001; Ronquist and Huelsenbeck 2003) on the CIPRES Web Portal (Miller et al. 2010) using best models. Two parallel MCMCMC runs were performed, each using four independent chains and 2 million generations for the mycobiont tree and 10 million generations for the photobiont tree, sampling every 1000^th^ tree. Tracer v. 1.6 (Rambaut and Drummond 2007) was used by plotting the log-likelihood values of the sample points against generation time. Convergence between runs was also verified using the Potential Scale Reduction Factor (PSRF) with all values equal or close to 1.000. Posterior Probabilities (PP) were determined by calculating a majority-rule consensus tree after discarding the initial 25% trees of each chain as the burn-in.

A Maximum Likelihood (ML) analysis was performed using RAxML-HPC2 v.8.2.10 (Stamatakis 2014) with 1000 ML bootstrap iterations (BS) and the GTRGAMMAI model for both analyses.

**Figure 1.**
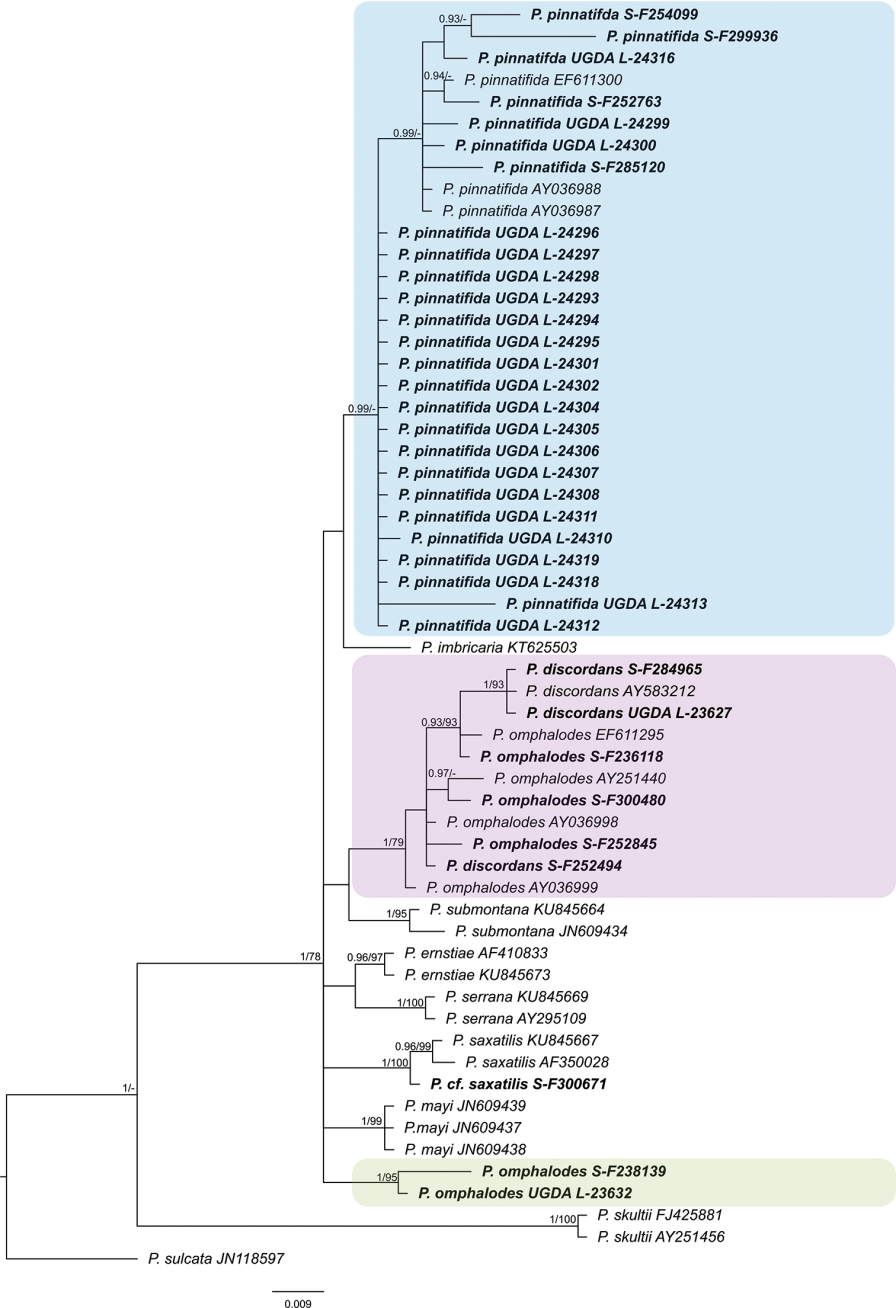
Phylogenetic relationships of *Parmelia discordans*, *P. omphalodes* and *P. pinnatifida*, based on Bayesian analysis of the ITS rDNA dataset. Posterior probabilities and maximum likelihood bootstrap values are shown near the internal branches. Newly generated sequences are described with herbarium numbers following the species names. GenBank Accession numbers of sequences downloaded from GenBank follow the species names. Clades with *Parmelia discordans*, *P. omphalodes* and *P. pinnatifida* are highlighted.

**Figure 2.**
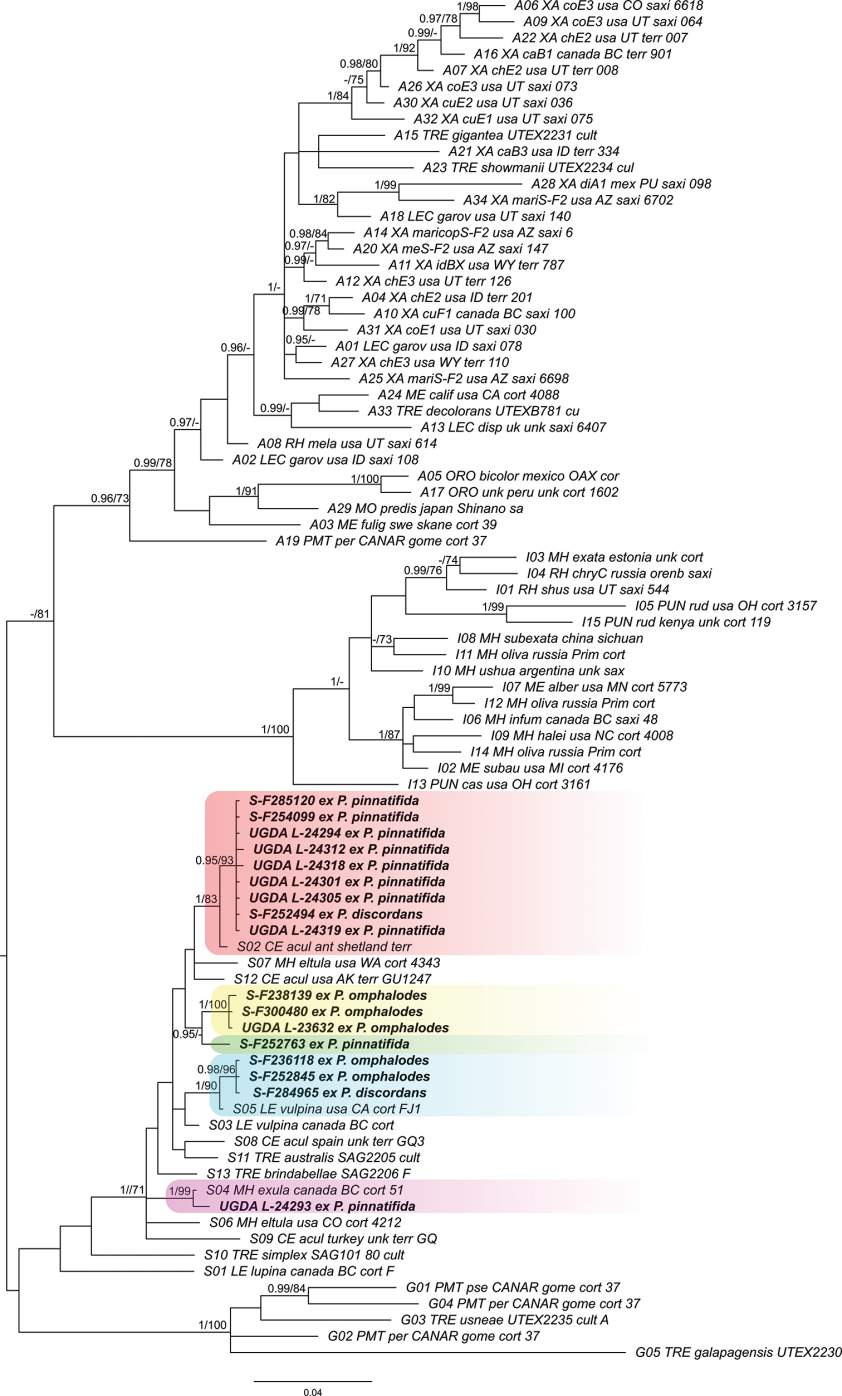
Phylogenetic placement of *Trebouxia* photobionts from selected *Parmelia* spp., based on Bayesian analysis of the ITS rDNA dataset. Posterior probabilities and maximum likelihood bootstrap values are shown near the internal branches. Newly generated sequences are in bold, with collecting numbers preceding the species names. Representative *Trebouxia* OTUs, as described in Leavitt et al. (2015), were downloaded from Dryad database (Dryad Digital Repository, Leavitt et al. 2015). Clades with photobionts from *Parmelia discordans*, *P. omphalodes* and *P. pinnatifida* are highlighted.

Phylogenetic trees were visualised using FigTree v. 1.4.2 (Rambaut 2012). Since the RAxML tree did not contradict the Bayesian tree topology for the strongly supported branches, only the latter was shown with the bootstrap support values, together with posterior probabilities of the Bayesian analysis (Figures 1, 2). BS ≥ 70 and PP ≥ 0.95 were considered to be significant and are shown near these branches.

### Haplotype network

**Figure 3.**
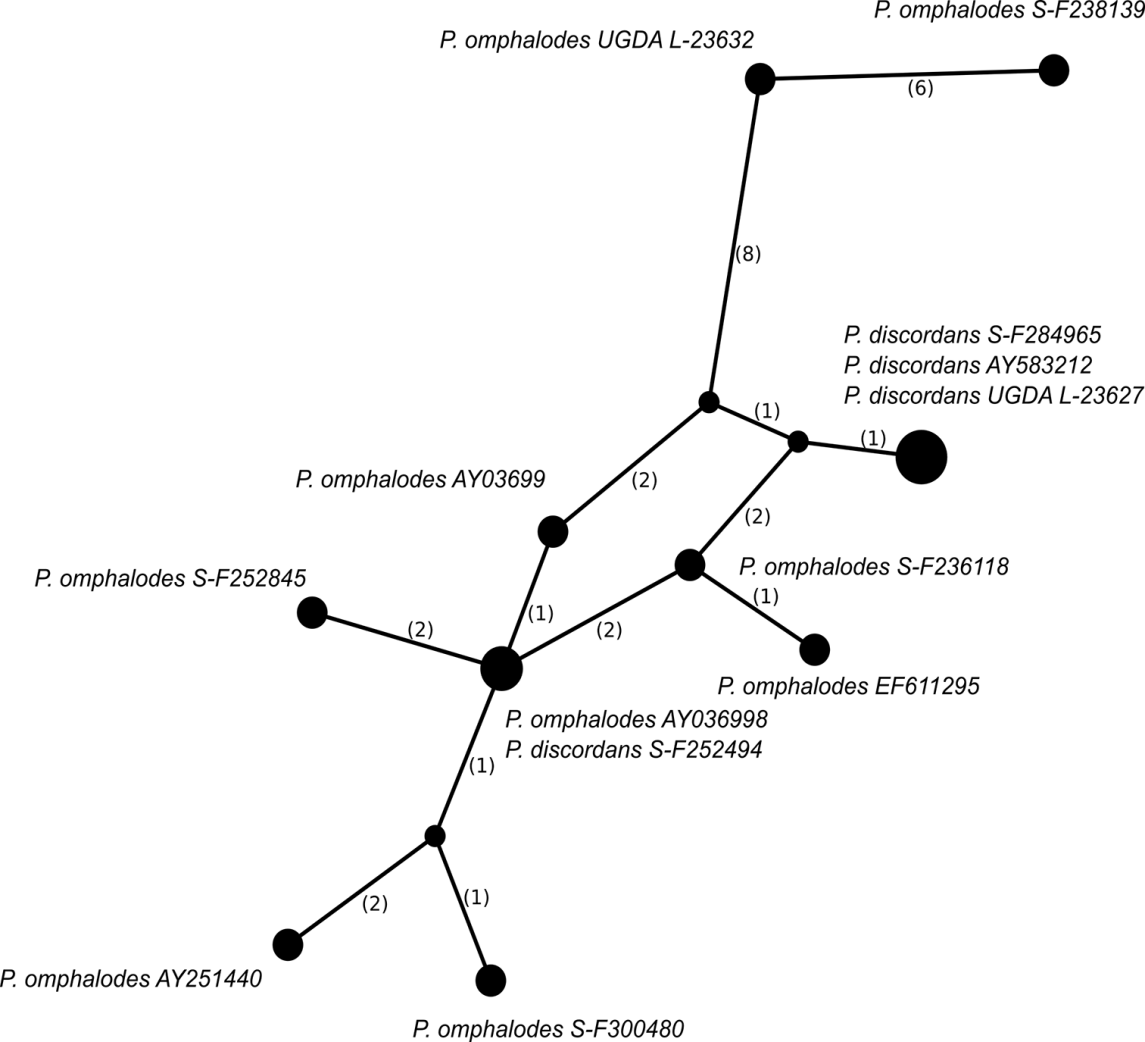
Haplotype network showing relationships between ITS rDNA sequences from *Parmelia discordans* and *P. omphalodes*. The names of species are followed with herbarium numbers of specimens or GenBank Accession Numbers. Mutational changes are presented as numbers in brackets near lines between haplotypes.

Sequences of ITS rDNA from specimens belonging to *P. discordans* and *P. omphalodes* were aligned using Seaview software (Galtier et al. 1996; Gouy et al. 2010) and the terminal ends were deleted. The alignment consisted of 13 sequences and 463 sites. The TCS network (Clement et al. 2002) was created using PopART software (http://popart.otago.ac.nz) (Figure 3).

### Niche similarity

To evaluate the similarity of niches occupied by all studied taxa, ecological niche modelling (ENM) was applied.

**Figure 4.**
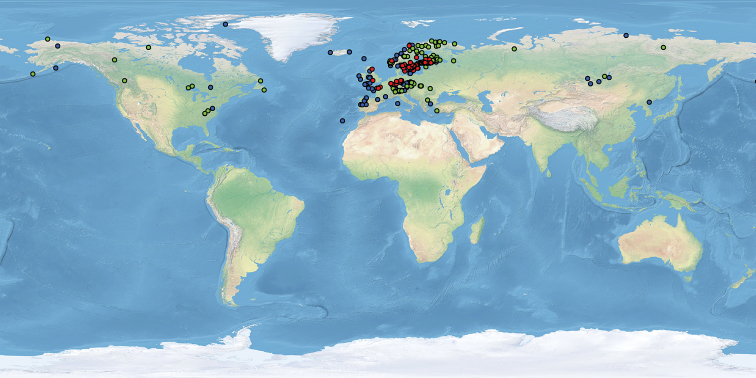
Localities of *Parmelia discordans* (red), *P. omphalodes* (blue) and *P. pinnatifida* (green) used in ENM analysis.

The database of localities of *P. discordans*, *P. omphalodes* and *P. pinnatifida* was compiled, based on information provided on labels of herbarium specimens. The geographic coordinates provided on the herbarium sheet labels were verified. If there were no information about the latitude and longitude on the herbarium sheet label, we followed the description of the collection site and assigned coordinates as precisely as possible to this location. Google Earth (Google Inc.) was used to validate all gathered information. In total, 61 records of *P. discordans*, 161 of *P. omphalodes* and 113 of *P. pinnatifida* were used to perform ENM analysis (Figure 4 and Suppl. material 1: Table S1).

The maximum entropy method, as implemented in Maxent version 3.3.2 software, was used to create models of the suitable niche distribution (Phillips et al. 2004, 2006). This application has been proved to provide the most robust response across the number of environmental variables tested (Duque-Lazo et al. 2016) and it has been shown to work better with a small number of samples than with other approaches (Hernandez et al. 2006). MaxEnt settings previously used in research where limited samples were available (e.g. Pietras and Kolanowska 2019) were used in our computations. To assess the high level of specificity of the analysis, the maximum iterations of the optimisation algorithm were established as 10000 and the convergence threshold as 0.00001. The neutral (= 1) regularisation multiplier value and auto features were used. The “random seed” option was used for selecting training points. The run was performed with 1000 bootstrap replications and the default logistic model was used. The Area Under the Receiver Operating Characteristic (AUC) was used to evaluate the reliability of analyses. This is a commonly used threshold independent metric for evaluation of species distribution models (Hosmer and Lemeshow 2000; Elith et al. 2006; Evangelista et al. 2008) which was also used in studies involving a small number of samples (Pietras and Kolanowska 2019). Using more specific metrics, which could evaluate the possible overfitting of the model, would require implementing absence points and, in the case of our study object, such a dataset could not be prepared due to the lack of comprehensive studies on the distribution of genus representatives.

Twelve bioclimatic variables in 2.5 minutes developed by Hijmans et al. (2005; http://www.worldclim.org) were used as input data (Table 3). The study area which was used to evaluate the global identity of niches occupied by *P. discordans*, *P. omphalodes* and *P. pinnatifida* extended from 86.583°N to 17.83°N. As some previous studies (Barve et al. 2011) indicated that usage of a restricted area in ENM analysis is more reliable than calculating habitat suitability on the global scale, the similarity of niches occupied in America was calculated for an area that extended from 180°W to 31.749°W and from 85.292°N to 17.833°N and the study area of all three species occurring in Eurasia was reduced to 84.83–17.83°N and 17.833°W-180°E.

**Table 3.** Variables used in the ENM analysis.

**bio1**	annual mean temperature
**bio2**	mean diurnal range (mean of monthly (max temp - min temp))
**bio3**	isothermality (mean diurnal range / temperature annual range * 100)
**bio4**	temperature seasonality (standard deviation *100)
**bio5**	max temperature of the warmest month
**bio8**	mean temperature of the wettest quarter
**bio12**	annual precipitation
**bio13**	precipitation of the wettest month
**bio14**	precipitation of the driest month
**bio15**	precipitation seasonality (coefficient of variation)
**bio18**	precipitation of the warmest quarter
**bio19**	precipitation of coldest quarter

The differences amongst the niches occupied by the populations of three studied lichens were evaluated using the niche identity indices: Schoener’s D (D) and I statistic (I) as available in ENMTools v1.3 (Schoener 1968; Warren et al. 2008, 2010). Additionally, the predicted niche occupancy (PNO) profiles were plotted to visualise differences in the preferred climatic factors amongst all taxa. PNO integrates species probability (suitability) distributions derived with MaxEnt with respect to a single climatic variable (Heibl and Calenge 2015).

Principal components analysis (PCA) was performed to explain the general variation pattern amongst the studied species, based on 12 bioclimatic factors used in ENM analysis. Statistical computations were performed with the programme PAST v. 3.0 (Hammer et al. 2001).

## Results and discussion

### Phylogeny, morphology and chemistry of species of *Parmelia omphalodes* group

Trees of similar topologies were generated using the maximum likelihood method (RaxML; best tree likelihood LnL = −1512.540166) and the Bayesian approach (BA; harmonic mean was −1667.09). The Bayesian tree is presented in Figure 1 with added bootstrap supports from the RaxML analysis and posterior probabilities from the BA. The phylogenetic analyses showed that, despite morphological similarities of species, the *P. omphalodes* group is not monophyletic. Specimens are separated into three distinct clades. One clade (0.99 PP) is related to *P. imbricaria* Goward et al. (Figure 1). In this clade, specimens containing salazinic acid, but variable in fatty and lobaric acids content (Table 2), are grouped with sequences labelled as *P. pinnatifida*, downloaded from GenBank. Analysis of morphological features revealed that all specimens in this clade have predominantly marginal pseudocyphellae. Specimens with similar chemical variation (Table 2), but having both marginal and laminal pseudocyphellae and, thus, referable to *P. omphalodes*, form two distinct clades (Figure 1), one containing the majority of the studied specimens and also the sequences downloaded from GenBank (1 PP and 79 BS) and the second (1 PP and 95 BS) grouping only two samples (specimens S-F238139 and UGDA L-23632). The latter clade consists of specimens indistinguishable in all morphological and chemical features from other specimens of *P. omphalodes* used in this study. This lineage may represent a cryptic species, but more specimens and additional molecular markers are necessary to be analysed before it is described.

Within the larger clade of *P. omphalodes*, four sequences obtained from specimens containing protocetraric acid and determined as *P. discordans* are nested. Three of those specimens form a highly supported lineage (1 PP and 93 BS), while the fourth sample of *P. discordans* is placed outside this subclade (Figure 1). Moreover, to better understand the phylogenetic position and genetic variation of the ITS rDNA marker within *P. omphalodes* s.l., we generated a haplotype network for specimens of both *P. discordans* and *P. omphalodes* (Figure 3). There is no significant difference between specimens of those two taxa, except two samples of *P. omphalodes* (specimens S-F238139 and UGDA L-23632) representing the second lineage found in our study (see above), that differ from other representatives of this species in at least 10 sites. One specimen of *P. discordans* (S-F252494) shares the same haplotype with *P. omphalodes* (AY036998), which differs from other haplotypes of the former taxon in 5 sites. Moreover, three other specimens of *P. discordans* share the same haplotype, which differs from haplotypes of *P. omphalodes* in at least 3 positions.

So far, the taxonomy of *P. omphalodes* group was unclear. Kurokawa (1976) recognised three species within this group: *P. discordans*, *P. omphalodes* and *P. pinnatifida*, whereas Skult (1984) classified *P. discordans* and *P. pinnatifida* as subspecies within *P. omphalodes*. On the other hand, Hale (1987) recognised two species, *P. discordans* and *P. omphalodes*. However, our results agree to a certain point with those presented by Molina et al. (2004) and Thell et al. (2008), who showed that *P. pinnatifida* is a taxon well-separated from *P. omphalodes*. In the case of *P. discordans*, Thell et al. (2008) used only a single sequence of this species (AY583212), which was nested within the *P. omphalodes* clade. In the discussion, those authors concluded that the status of *P. discordans* as a separate taxon required further molecular analyses (Thell et al. 2008). In our study, sequences of *P. discordans* are also nested in the clade of *P. omphalodes*. Perhaps the former should be synonymised with *P. omphalodes*, as some specimens of both taxa share the same ITS rDNA haplotypes (Figure 3). However, the final conclusions should await more data from other molecular markers as the use of a single genetic marker to delimit species might be inappropriate (e.g. Leavitt et al. 2011, 2013a; Pino-Bodas et al. 2013). However, in the case of many taxonomic groups, ITS rDNA helps to discriminate species, for example, in *Parmeliaceae*, including *Parmelia*, and has been shown to be effective and proposed to be used as a primary fungal barcode (e.g. Crespo and Lumbsch 2010; Leavitt et al. 2014; Divakar et al. 2016; Corsie et al. 2019).

**Figure 5.**
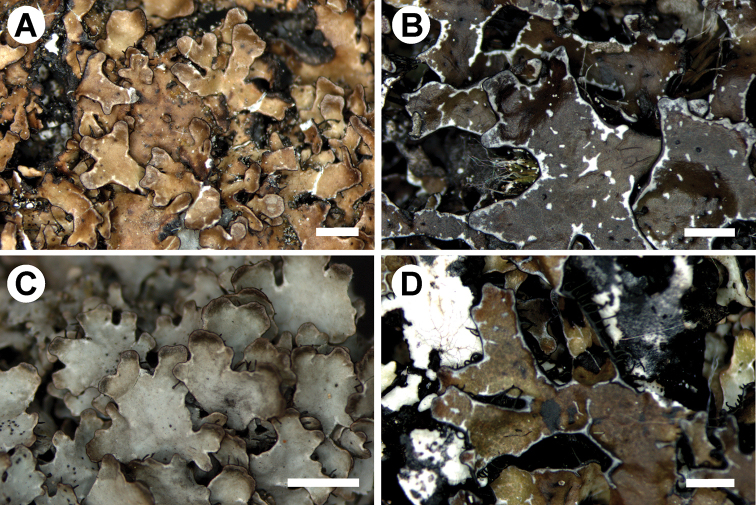
**A***Parmelia discordans*, with marginal and laminal pseudocyphellae, laminal pseudocyphellae mostly not connected with marginal ones (S F-252494) **B***P. omphalodes*, with marginal and laminal pseudocyphellae, laminal pseudocyphellae mostly not connected with marginal ones (S F-252845) **C***P. pinnatifida*, with marginal pseudocyphellae (UGDA L-24298) **D***P. pinnatifida*, with marginal and laminal pseudocyphellae, laminal pseudocyphellae starting predominantly from pseudocyphellae formed at the edge of lobes (S F-239397). Scale bars: 200 μm (**A, B, D**), 150 μm (**C**).

The distinguishing character between *P. omphalodes* and *P. pinnatifida* is the development of pseudocyphellae; however, the determination of the type and orientation of pseudocyphellae requires checking of the entire thallus surface, not only marginal or central parts of the thalli. We concluded that *P. pinnatifida* has mostly marginal pseudocyphellae forming white rims around lobes margins (Figure 5C), in some samples with few laminal ones in older parts of thalli. Laminal pseudocyphellae, in this species, predominantly start at the edge of lobes and are connected to the marginal pseudocyphellae and only very few are separated from the marginal ones (Figures 5C, D). Thalli of *P. omphalodes* always have marginal and laminal pseudocyphellae and, in the case of the latter, many are not connected to the margins of lobes (Figure 5B). We also checked the orientation of pseudocyphellae in *P. discordans*. In young thalli, they may be exclusively marginal, but in most cases laminal ones are also developed (Figure 5A), as in the case of *P. omphalodes*.

The presence of lobaric and fatty acids cannot be treated as diagnostic for the separation of *P. omphalodes* and *P. pinnatifida*, as it does not correspond with molecular data. Until now, *P. pinnatifida* was characterised as a species lacking lobaric acid (Kurokawa 1976; Skult 1984; Molina et al. 2004; Ossowska and Kukwa 2016). In this study, the specimens with morphology of pseudocyphellae typical for this species and with or without lobaric acid are grouped in one clade. The same variation in the presence of lobaric acid was noted in *P. omphalodes*, which was reported as constantly containing this substance (Kurokawa 1976; Skult 1984; Ossowska and Kukwa 2016). A similar issue was noted in the *P. saxatilis* group. The presence or absence of lobaric acid was treated as a diagnostic character to differentiate species (e.g. Feuerer and Thell 2002; Molina et al. 2004; Thell et al. 2011; Ossowska et al. 2014), but the recent results obtained by Thell et al. (2017), Ossowska et al. (2018), Corsie et al. (2019) and Haugan and Timdal (2019), revealed that the production of this substance is variable, for example, *P. serrana* A. Crespo et al., typically lacking lobaric acid, may also produce this substance (Ossowska et al. 2018; Corsie et al. 2019; Haugan and Timdal 2019). Similar variation in lobaric acid production was also observed in *Stereocaulon condensatum* Hoffm. (Oset 2014). Moreover, lobaric acid was detectable in *P. omphalodes* and *P. pinnatifida* only when lobes from the central parts of the thalli were taken for TLC.

Kurokawa (1976) reported that *P. omphalodes* and *P. pinnatifida* also differ in the production of fatty acids (absent in *P. omphalodes*, present in *P. pinnatifida*), but both species also showed intraspecific variation in this character (Table 2). Moreover, the detection of fatty acids may differ due to the type of TLC plates used. The glass TLC plates are better suited for the detection of these substances than aluminium plates (Orange et al. 2001) and, for example, protolichesterinic acid was undetectable on aluminium plates, but visible on glass plates.

Morphological and chemical characteristics of all taxa of the group are summarised in Table 4 and the determination key is presented below (see also Table 2).

**Table 4.** Historical and present overview of species delimitations within the *Parmelia omphalodes* group with their morphological and chemical characteristics (ATR – atranorin, SAL – salazinic acid with consalazinic acid, LOB – lobaric acid, PRC – protocetraric acid, PRL – protolichesterinic acid, FAT – fatty acids; + present in all specimens; ± sometimes present).

	**Taxa**	**Morphology**	**Chemistry**
**Kurokawa (1976)**	*P. discordans*	pseudocyphellae marginal and laminal; lobules absent; lobes 1–2.5 mm wide	ATR (+), PRC (+), LOB (+), FAT (±)
	*P. omphalodes*	pseudocyphellae marginal and laminal; lobules present	ATR (+), SAL (+), LOB (+)
	*P. pinnatifida*	pseudocyphellae marginal; narrow lobules present; lobes repeatedly branched	ATR (+), SAL (+), FAT (+)
**Skult (1984)**	P. omphalodes subsp. discordans	pseudocyphellae sparse and marginal in young lobes; lobes diameter 0.13–2.8 mm	ATR (+), PRC (+), LOB (+), PRL (+)
	P. omphalodes subsp. omphalodes	pseudocyphellae marginal and laminal; lobes up to 3.5 mm diameter	ATR (+), SAL (+), LOB (+), PRL (±)
	P. omphalodes subsp. pinnatifida	pseudocyphellae marginal, in old lobes laminal; lobes narrow, 0.13–2.9 mm diameter	ATR (+), SAL (+), PRL (±)
**Hale (1987)**	*P. discordans*	pseudocyphellae marginal, few also laminal; lobes 1–3 mm wide	ATR (+), PRC (+), LOB (+), unidentified FAT (±)
	*P. omphalodes*	pseudocyphellae mostly marginal; lobes wide 1–4 mm	ATR (+), SAL (+), LOB (±), PRL (+)*
**Molina et al. (2004)**	*P. discordans*	pseudocyphellae linear; lobes overlapping, 1–3 mm wide	PRC (+), LOB (+)
	*P. omphalodes*	lobes 4 mm wide	ATR (+), SAL (+), LOB (+), PRC (±)
	*P. pinnatifida*	pseudocyphellae restricted to the margins; lobes narrow, repeatedly branched and overlapping	ATR (+), SAL (+), PRL (+)
**Thell et al. (2008)**	*P. discordans*	pseudocyphellae indistinct; lobes narrow	ATR (+), PRC (+), LOB (+)
	*P. omphalodes*	–	ATR (+), SAL (+), LOB (+), PRL (+), PRC (±)
	*P. pinnatifida*	pseudocyphellae marginal; lobes narrow	ATR (+), SAL (+), PRL (+), PRC (±)
**This study**	*P. discordans*	pseudocyphellae marginal and laminal, laminal pseudocyphellae at least partly not starting from the lobe margins; lobes narrow and sublinear, about 1–3 mm wide and 1–3 mm length	ATR (+), PRC (+), LOB (±), FAT (±)
	*P. omphalodes*	pseudocyphellae marginal and laminal, laminal pseudocyphellae mostly not starting from the lobe margins; lobes broad and sublinear, about 2–3 mm wide and 3–4 mm length	ATR (+), SAL (+), LOB (±), FAT (±)
	*P. pinnatifida*	pseudocyphellae marginal, in older parts of thalli with few laminal connected to the lobes margins; lobes narrow, sublinear, about 1–2 mm wide and 0.5–2 mm length	ATR (+), SAL (+), LOB (±), FAT (±)

* Author described the lack of lobaric acid in 96% of analysed samples, but morphologically they were similar to *P. omphalodes*. Hale (1987) did not classified them as a *P. pinnatifida*.

### Phylogenetic analyses of photobionts

**Figure 6.**
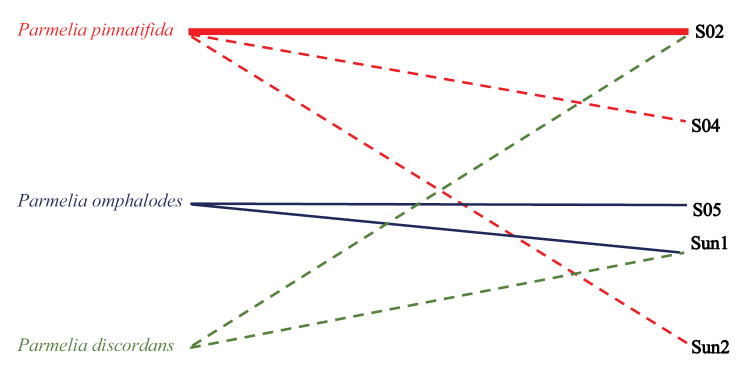
Association network between lichen mycobionts of *P. omphalodes* group (i.e. *Parmelia discordans*, *P. omphalodes* and *P. pinnatifida*) and photobiont OTUs. The line width is proportional to the number of specimens forming the association with the particular OTU. SUn1 and SUn2 represent unnamed lineages of *Trebouxia* belonging to clade S.

Trees of similar topologies were generated using maximum likelihood (RaxML; best tree likelihood LnL = -7013.073328) and Bayesian analysis (BA; harmonic mean was -6996.31). The Bayesian tree is presented in Figure 2 with added bootstrap supports from RaxML and posterior probabilities from BA. The phylogenetic analyses showed that photobionts of *P. discordans*, *P. omphalodes* and *P. pinnatifida* belong to the *Trebouxia* S clade (*T. simplex*/*letharii*/*jamesii* group) sensu Leavitt et al. (2015) and represent at least five different lineages (Figure 2). The most common photobiont in the species analysed in this work is *Trebouxia* OTU S02, which was found in one specimen of *P. discordans* and most specimens of *P. pinnatifida* (Figure 6). Additionally, we detected *Trebouxia* OTU S04 in a single specimen of *P. pinnatifida* (UGDA L-24293) and one specimen of this species (S-F252763) has an unnamed *Trebouxia* species (SUn2). Therefore, *P. pinnatifida* associates with at least three different photobiont taxa of which, based on the BLAST search, OTU S04 seems to be very rare. We also found some variation in photobionts of *P. omphalodes* which associates with two lineages of *Trebouxia*, i.e. OTU S05 (two specimens) and an unnamed *Trebouxia* lineage (three specimens) (SUn1), closely related to the photobiont present in one sample of *P. pinnatifida* (S-F252763). Moreover, *Trebouxia* OTU S05 was also detected in *P. discordans*. In Leavitt et al. (2015), it was reported that, based on 98% sequence similarity, *Parmelia* species form associations with *Trebouxia* OTU I02, belonging to the *T. impressa/galapagensis* group, but this group of photobionts might only be characteristic for *P. saxatilis* and *P. sulcata* groups, as we have not found this lineage in the studied specimens.

According to Beck et al. (2002), ‘selectivity’ refers to the taxonomic range of partners that are selected by one of the bionts, while ‘specificity’ should be used for the symbiotic association and depends on the range and taxonomic relatedness of acceptable partners. Lichens with high selectivity may associate with a limited number of photobionts. Numerous mycobionts, belonging to Parmeliaceae, have been shown to associate with identical species of *Trebouxia*, while others exhibited higher photobiont flexibility (e.g. Kroken and Taylor 2000; Ohmura et al. 2006, 2018; Doering and Piercey-Normore 2009; Leavitt et al. 2013b, 2015; Lindgren et al. 2014). Our results indicate that taxa from *P. omphalodes* group are moderately selective in their photobionts choice, as these taxa associate with at least two or three *Trebouxia* lineages (Figure 6).

Lichens that reproduce sexually via independent dispersal of fungal spores, undergo a process of re-lichenisation. This means that the germinating spore of the mycobiont can easily exchange its autotrophic partner, in contrast to asexually reproducing lichens distributing both partners together, which allows continuation of the symbiosis without the need to re-associate with another biont (Beck et al. 1998, 2002; Romeike et al. 2002; Sanders and Lücking 2002). However, even asexually reproducing lichens, such as the *Lepraria* species, have been shown to switch their algal partners (Nelsen and Gargas 2008). Moreover, in populations of *Physconia grisea* (Lam.) Poelt with a vegetative propagation strategy, mycobionts associate with more than one photobiont genotype (Wornik and Grube 2010). It was also reported that both sexual and vegetative reproduction allows lichens to generate almost the same amount of diversity to adapt to their environments (Cao et al. 2015). Moreover, *Protoparmeliopsis muralis* (Schreb.) M.Choisy, which does not produce vegetative propagules, exhibited a low selectivity level (Guzow-Krzemińska 2006; Muggia et al. 2013); however, *P. muralis* has wider geographical distribution and occurs on a wider range of substrata and ecological conditions than taxa from the analysed group.

The ecological ’lichen guilds‘ hypothesis, i.e. communities of lichens growing on the same type of habitat and forming associations with the same photobiont species, have been proposed for cyanobacterial lichens (Rikkinen et al. 2002). This hypothesis was tested by Peksa and Škaloud (2011) for the eukaryotic genus *Asterochloris* Tschermak-Woess. These authors showed that ecological niches available to lichens may be limited by algal preferences for environmental factors and thus can lead to the existence of specific lichen guilds, but their results were based only on selected species of *Lepraria* Ach. and *Stereocaulon* Hoffm. On the other hand, results obtained by Leavitt et al. (2015) indicated that ecologically specialised lichens from different genera form associations with different *Trebouxia* OTUs in the same habitat. Moreover, observations made by Deduke and Piercey-Normore (2015) for species of *Xanthoparmelia* (Vain.) Hale, growing on different rock types, did not support the photobiont guild hypothesis. However, they suggested that the range of rock substrata type in their study may have been too narrow to differentiate algal preference. On the other hand, they indicated that Peksa and Škaloud (2011) compared broadly defined types of substrata (defined as a ‘bark of tree’ and ‘rock’).

In this study, we found that the most common photobiont in *P. pinnatifida* was *Trebouxia* OTU S02. All samples of *P. pinnatifida* were collected from rocks; however, some authors previously reported the same *Treboxia* OTU S02 from terricolous, saxicolous and corticolous Parmeliaceae (i.e. genera *Cetraria* Ach., *Melanohalea* O.Blanco et al., *Montanelia* Divakar et al., *Protoparmelia* M.Choisy and *Rhizoplaca* Zopf and species *Xanthoparmelia coloradoensis* Hale and *Vulpicida juniperinus* (L.) J.-E.Mattsson & M.J.Lai) (Lindgren et al. 2014; Leavitt et al. 2015; Singh et al. 2017), but it may also occur in lichen genera representing other families, for example, *Chaenotheca* (Th.Fr.) Th.Fr., *Circinaria* Link and *Umbilicaria* Hoffm. (Beck 2002; Romeike et al. 2002; Molins et al. 2018). On the other hand, *Trebouxia* OTU S04, which corresponds to *T. jamesii* (UBT-86.156C3), was identified in a single specimen of *P. pinnatifida* (UGDA L-24293). It was previously reported exclusively from corticolous *Melanohalea* and *Bryoria* species (Lindgren et al. 2014; Leavitt et al. 2015) and seems to be very rare or at least rarely sampled, as it is poorly represented in GenBank. Moreover, the unnamed lineage of *Trebouxia* (SUn2) was detected in a single specimen of *P. pinnatifida* and, based on 99% identity, we found that it may also associate with, for example, *Bryoria simplicior* (Vain.) Brodo & D.Hawksw., *Cetraria aculeata* (Schreber) Fr., *Evernia divaricata* L. (Ach.) (Piercey-Normore 2009; Domaschke et al. 2012; Lindgren et al. 2014). Some variation in photobionts was also found in specimens of *P. omphalodes* which associate with *Trebouxia* OTU S05 and an unnamed lineage (SUn1). Leavitt et al. (2015) reported *Trebouxia* OTU S05, which corresponds to *Trebouxia suecica* (SAG2207), from terricolous and corticolous Parmeliaceae (i.e. *Cetraria aculeata* (Schreber) Fr., *Letharia vulpina* (L.) Hue and *Melanohalea* spp.). Photobionts, very similar to *Trebouxia* OTU S05 (100% identity), were additionally found in, for example, *Bryoria fremontii* (Tuck.) Brodo & D.Hawksw., *Lasallia hispanica* (Frey) Sancho & Crespo, *Lecanora rupicola* (L.) Zahlbr. and *Tephromela atra* (Huds.) Hafellner (Blaha et al. 2006; Lindgren et al. 2014; Muggia et al 2014; Paul et al. 2018). Moreover, the unnamed lineage of *Treboxia* (SUn1) was detected in three specimens of *P. omphalodes* and, based on 99% identity, we found that it may also associate with, for example, *Bryoria* spp., *Cetraria* spp., *Evernia mesomorpha* Nyl. *Flavocetraria nivalis* (L.) Kärnefelt & A.Thell and *Vulpicida pinastrii* (Scop.) J.-E.Mattsson & M.J.Lai (Opanowicz and Grube 2004; Piercey-Normore 2009; Lindgren et al. 2014; Onuț-Brännström et al. 2018). Therefore, the results obtained, based on our dataset, do not support the ecological guild hypothesis; however, our sampling was rather limited and we did not analyse co-occurring species. Although the type of substrata seems not to correspond to any of *Trebouxia* OTUs, bioclimatic factors, such as annual mean temperature, maximum temperature of warmest month or precipitation, may influence the patterns of photobionts distribution. However, to perform such an analysis, a larger set of specimens should be examined.

Interestingly, although *P. omphalodes* was found to associate with two lineages of *Trebouxia* photobionts (i.e. OTU S05 and an unidentified lineage SUn1), it does not associate with *Trebouxia* OTU S02, which, on the other hand, was found to associate with *P. discordans* (two samples). However, *P. discordans* also associates with *Trebouxia* OTU S05. As those species differ in morphology and chemistry, we suggest that those differences might be related to the photobiont type. Although some researchers did not find any correlation between different chemotypes and the associated photobionts (e.g. Blaha et al. 2006; Lindgren et al. 2014), recent studies suggested that the production of certain secondary metabolites might be triggered by the environment, for example, climate, edaphic factors or associated symbionts (e.g. Spribille et al. 2016; Lutsak et al. 2017). However, due to limited sampling, we cannot confirm this hypothesis for *Parmelia* spp. analysed in this study.

### Ecological niche modelling of species of *Parmelia omphalodes* group

**Figure 7.**
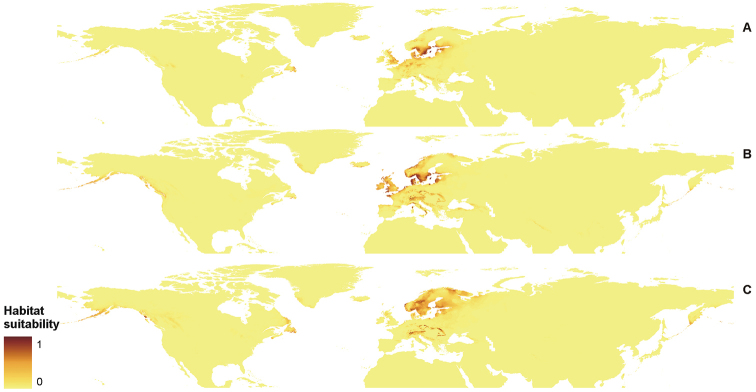
Distribution of suitable niches of *P. discordans* (**A**), *P. omphalodes* (**B**) and *P. pinnatifida* (**C**) in the Northern Hemisphere.

**Figure 8.**
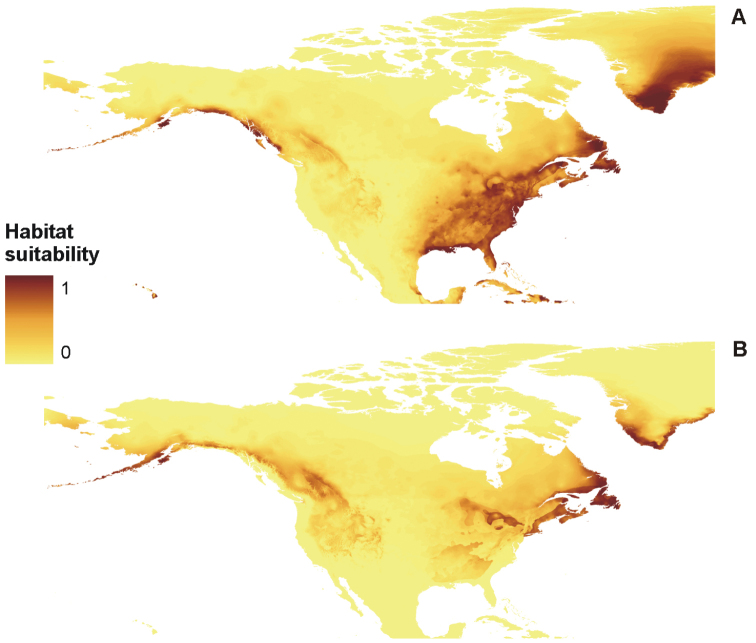
Distribution of suitable niches of *P. omphalodes* (**A**) and *P. pinnatifida* (**B**) in America.

**Figure 9.**
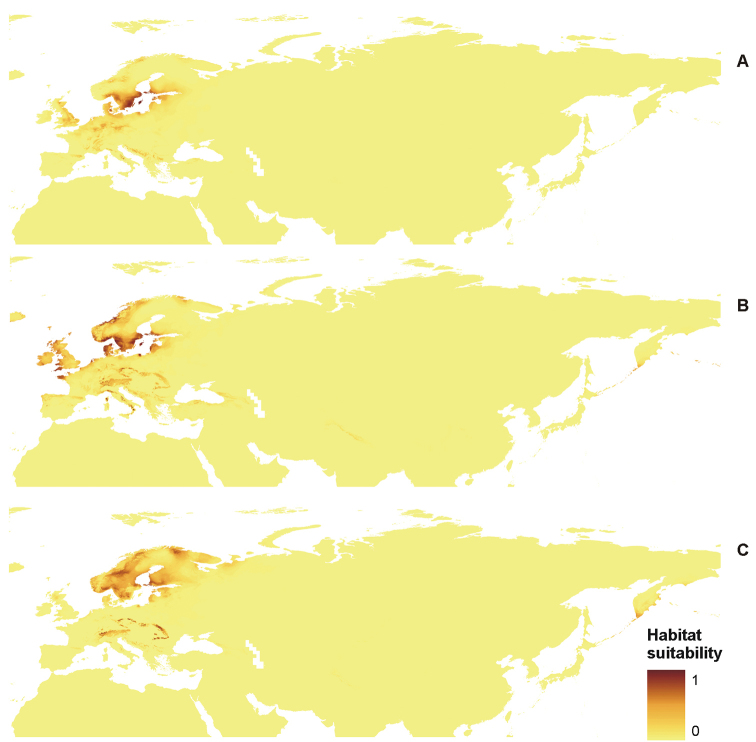
Distribution of suitable niches of *P. discordans* (**A**), *P. omphalodes* (**B**) and *P. pinnatifida* (**C**) in Eurasia.

The created models, derived from MaxEnt, received high AUC scores, indicating high reliability of analyses (Table 5). Generated maps of distribution of suitable niches of the three lichen species were wider than the known geographical range of these lichens (Figures 7–9).

**Table 5.** The average training AUC for created models.

	**Northern Hemisphere**	**Eurasia**	**America**
***P. discordans***	0.993 (SD = 0.001)	0.992 (SD = 0.001)	–
***P. omphalodes***	0.980 (SD = 0.003	0.982 (SD = 0.002)	0.767 (SD = 0.101)
***P. pinnatifida***	0.981 (SD = 0.003	0.986 (SD = 0.002)	0.819 (SD = 0.064)

The distribution of *P. discordans* is limited mainly by precipitation of the driest month (bio14), but two other factors that can influence the occurrence of this taxon, varied in analyses conducted for the Northern Hemisphere and Eurasia separately. While in the former analysis, annual mean temperature (bio1) and mean diurnal range (bio2) gave important contributions to the model, the latter analysis indicated maximum temperature of the warmest month (bio5) and temperature seasonality (bio4) as significant limiting factors. Additionally, in cases of *P. omphalodes* and *P. pinnatifida*, different variables gave various contributions to the models created for different study areas. Mean diurnal range (bio2) was the crucial limiting factor for Eurasian populations of *P. omphalodes*, while within the American range of this species, its occurrence depends on precipitation of the driest month (bio14). For the American distribution of *P. pinnatifida*, the annual mean temperature (bio1) significantly influenced the model and the distribution of Eurasian populations appears limited by the maximum temperature of the warmest month (bio5) (Table 6).

**Table 6.** Estimates of relative contributions of the environmental variables to the Maxent model.

	**Northern Hemisphere**	**Eurasia**	**America**
***P. discordans***	bio14 (25.6)	bio14 (35.9)	–
	bio1 (18.8)	bio5 (15.2)	
	bio2 (15.4)	bio4 (14.6)	
***P. omphalodes***	bio19 (21.1)	bio2 (27.8)	bio14 (48.2)
	bio4 (21)	bio19 (24.8)	bio15 (20.3)
	bio2 (17.7)	bio4 (14.2)	bio2 (10.9)
***P. pinnatifida***	bio5 (17.7)	bio5 (24.6)	bio1 (42.2)
	bio14 (17.3)	bio14 (19.1)	bio14 (18)
	bio4 (14.1)	bio4 (15.7)	bio8 (11.1)

**Figure 10.**
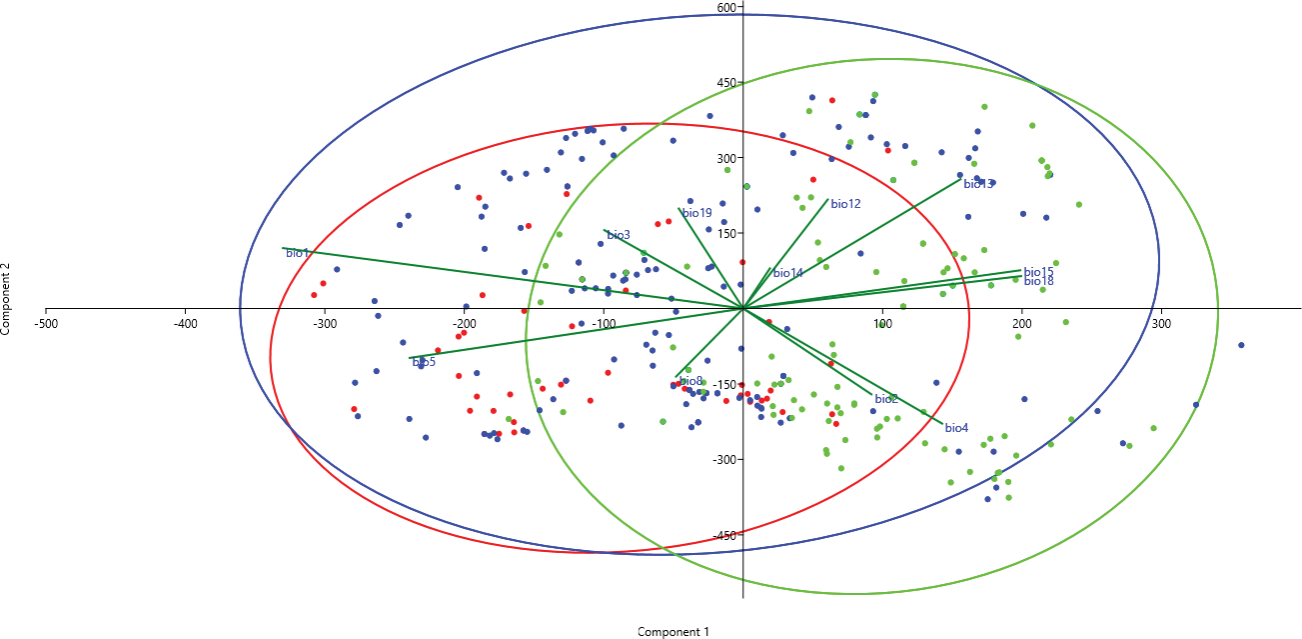
Principal components analysis (PCA) of *P. discordans* (red), *P. omphalodes* (blue) and *P. pinnatifida* (green), based on the bioclimatic factors from individuals.

The PCA diagram (Figure 10) showed that the highest bioclimatic variation is observed in *P. omphalodes* and that niches of *P. discordans* and *P. pinnatifida* are embedded in this highly flexible bioclimatic tolerance of *P. omphalodes*. The overall high similarity in bioclimatic preferences of all three studied taxa is presented in PNO profiles created for various geographic areas (Suppl. material 2: Figure S2, Suppl. material 3: Figure S3, Suppl. material 4: Figure S4). On a global scale, *P. pinnatifida* and *P. omphalodes* occupy similar niches (D = 0.581, I = 0.840), while bioclimatic preferences of *P. discordans* are more similar to *P. omphalodes* than to *P. pinnatifida* (Table 7). In the American range, *P. omphalodes* and *P. pinnatifida* occupy very similar habitats (D = 0.821, I = 0.968; Table 8). Within Eurasian populations, the highest similarity is observed for *P. omphalodes* and *P. discordans* (D = 0.587, I = 0.828); however, *P. pinnatifida* and *P. omphalodes* also occupy similar niches (D = 0.564, I = 0.820; Table 9).

**Table 7.** Niche identity indexes calculated for Northern Hemisphere.

**D\I**	***P. discordans***	***P. omphalodes***	***P. pinnatifida***
***P. discordans***	x	0.791	0.703
***P. omphalodes***	0.544	x	0.840
***P. pinnatifida***	0.441	0.581	x

**Table 8.** Niche identity indexes calculated for America.

**D\I**	***P. omphalodes***	***P. pinnatifida***
***P. omphalodes***	x	0.968
***P. pinnatifida***	0.821	x

**Table 9.** Niche identity indexes calculated for Eurasia.

**D\I**	***P. discordans***	***P. omphalodes***	***P. pinnatifida***
***P. discordans***	x	0.828	0.729
***P. omphalodes***	0.587	x	0.820
***P. pinnatifida***	0.468	0.564	x

According to published data (Sanders and Lücking 2002; Büdel and Scheidegger 2008), lichens without vegetative propagules, dispersing both bionts independently, require the contact of the mycobiont with a compatible photobiont species in suitable environmental conditions to establish new thalli. Results of ecological niche modelling, presented here, confirmed that species from the analysed group occupy similar niches. In Figure 2, one sequence of photobionts, associating with *P. discordans*, belong to *Trebouxia* OTU S05 and the second to *Trebouxia* OTU S02. The latter is the most common photobiont of *P. pinnatifida* which, on the other hand, was also found to associate with *Trebouxia* OTU S04 and an unnamed *Trebouxia* lineage SUn2. However, none of photobionts from *P. omphalodes* belongs to *Trebouxia* OTU S02 and OTU S04, but this taxon associates with two lineages of *Trebouxia* photobionts (i.e. OTU S05 and an unnamed lineage SUn1). These results show that, despite the species from *P. omphalodes* group differing in associated photobiont species, they exhibit similar niche preferece.

PCA (Figure 10) results showed that *P. omphalodes* is characterised by the highest bioclimatic variation in comparison with other species from the *P. omphalodes* group. On the other hand, the ENM method has shown that the potential distribution of *P. omphalodes* is wider than its known current occurrence range (Figures 4, 6–8). The absence of this taxon in the potential niches may be caused by the lack of suitable photobiont species in those areas or that the model did not capture the relevant variation and so overestimates the niche. Two *Trebouxia* lineages are found in this species, i.e. OTU S05 and an unnamed lineage. Such flexibility in the photobiont choice may facilitate the mycobiont colonisation of new niches; however, some of those photobionts may be relatively rare. *Trebouxia* OTU S05, which corresponds to the generalist *Trebouxia suecica*, was previously reported from numerous terricolous and corticolous species in temperate, boreal and alpine climates, while the unnamed lineage of *Trebouxia* (SUn1, Table 10), present in three specimens, probably also occurs in selected terricolous and corticolous species (Table 10). Probably the latter is characterised by narrower ecological amplitude, but it needs further studies. On the other hand, *P. pinnatifida* forms associations with three *Trebouxia* lineages, i.e. OTUs S02 and S04 and an unnamed lineage (SUn2, Table 10). Most photobiont sequences from *P. pinnatifida* were grouped in OTU S02 clade. They were collected from different localities in Poland (Beskidy Mts, Sudety Mts, Stołowe Mts), Norway and Sweden. Moreover, the same *Trebouxia* OTU S02 was found in terricolous, saxicolous and corticolous lichens (e.g. Leavitt et al. 2015). It suggests that *Trebouxia* OTU S02 has a broad ecological amplitude and worldwide distribution. Therefore *P. pinnatifida* may also have wider geographical distribution than current data suggest. The absence of those species in some localities may be caused by the lack of unambiguous morphological and chemical features necessary for their identification. For this reason, herbarium material from the group *P. omphalodes* requires re-determination. On the other hand, the possible overestimation of the MaxEnt models may be due to additional, ecological factors (e.g. interaction with other organisms) which were not included in our analyses, but limit the distribution of the studied lichens.

**Table 10.** *Trebouxia* OTUs associating with species from *P. omphalodes* group with the information about their distribution, substrata preferences and references.

**OTUs**	**Distribution**	**Substrata**	**References**
**S02**	Antarctica, Austria, Canada, Chile, Germany, Greenland, Iceland, Morocco, Norway, Poland, Portugal, Russia, Slovakia, Spain, Sweden, UK, USA	corticolous, saxicolous and terricolous	Muggia et al. 2014, Leavitt et al. 2015, Singh et al. 2017, this study
**S04**	Canada, Estonia, Germany, Netherlands, Poland, Sweden, Turkey, USA	corticolous and saxicolous	Leavitt et al. 2015, this study
**S05**	Canada, Finland, Italy, Norway, Spain, Sweden, Turkey, USA	corticolous, saxicolous and terricolous	Blaha et al. 2006, Muggia et al. 2014, Leavitt et al. 2015, Singh et al. 2017, Dal Grande et al. 2018, Paul et al. 2018, this study
**SUn1**	Canada, Finland, Spain, Sweden	corticolous and terricolous	Opanowicz and Grube 2004, Piercey-Normore 2009, Lindgren et al. 2014, Onuț-Brännström et al. 2018, this study
**SUn2**	Canada, Norway, Russia, Sweden	corticolous and terricolous	Piercey-Normore 2009, Domaschke et al. 2012, Lindgren et al. 2014, this study

### Key to *Parmelia species* from the non-vegetative propagules group

**Table:** 

1	Pseudocyphellae marginal	**2**
–	Pseudocyphellae marginal and laminal (at least in older parts of thalli)	**3**
2	Salazinic acid present	***P. pinnatifida***
–	Protocetraric acid present	***P. discordans* (young thalli, rare)**
3	Lobes 0.5–2 mm long and 1–2 mm wide, laminal pseudocyphellae predominantly connected with marginal pseudocyphellae, very few pseudocyphellae not starting from the lobe edges	***P. pinnatifida***
–	Lobes 1–4 mm long and 1–3 mm wide, laminal pseudocyphellae predominantly not connected to the lobe margins	**4**
4	Protocetraric present	***P. discordans***
–	Salazinic acid present	***P. omphalodes***

## Supplementary materials

Supplementary material 1 Table S1. Database of localities used in the analyses with the bioclimatic values for each record Data type: occurrence https://binary.pensoft.net/file/364142This dataset is made available under the Open Database License (http://opendatacommons.org/licenses/odbl/1.0/). The Open Database License (ODbL) is a license agreement intended to allow users to freely share, modify, and use this Dataset while maintaining this same freedom for others, provided that the original source and author(s) are credited.Emilia Ossowska, Beata Guzow-Krzemińska, Marta Kolanowska, Katarzyna Szczepańska, Martin Kukwa

Supplementary material 2 Figure S2. PNO profiles created for *P. discordans* (A), *P. omphalodes* (B) and *P. pinnatifida* (C) in Northern Hemisphere Data type: multimedia https://binary.pensoft.net/file/364143This dataset is made available under the Open Database License (http://opendatacommons.org/licenses/odbl/1.0/). The Open Database License (ODbL) is a license agreement intended to allow users to freely share, modify, and use this Dataset while maintaining this same freedom for others, provided that the original source and author(s) are credited.Emilia Ossowska, Beata Guzow-Krzemińska, Marta Kolanowska, Katarzyna Szczepańska, Martin Kukwa

Supplementary material 3 Figure S3. PNO profiles created for *P. discordans* (A), *P. omphalodes* (B) and *P. pinnatifida* (C) in Eurasia Data type: multimedia https://binary.pensoft.net/file/364144This dataset is made available under the Open Database License (http://opendatacommons.org/licenses/odbl/1.0/). The Open Database License (ODbL) is a license agreement intended to allow users to freely share, modify, and use this Dataset while maintaining this same freedom for others, provided that the original source and author(s) are credited.Emilia Ossowska, Beata Guzow-Krzemińska, Marta Kolanowska, Katarzyna Szczepańska, Martin Kukwa

Supplementary material 4 Figure S4. PNO profiles created for *P. omphalodes* (A) and *P. pinnatifida* (B) in America Data type: multimedia https://binary.pensoft.net/file/364145This dataset is made available under the Open Database License (http://opendatacommons.org/licenses/odbl/1.0/). The Open Database License (ODbL) is a license agreement intended to allow users to freely share, modify, and use this Dataset while maintaining this same freedom for others, provided that the original source and author(s) are credited.Emilia Ossowska, Beata Guzow-Krzemińska, Marta Kolanowska, Katarzyna Szczepańska, Martin Kukwa
