# Supplementary material for: Morphology and secondary chemistry in species recognition of Parmelia omphalodes group – evidence from molecular data with notes on the ecological niche modelling and genetic variability of photobionts
Source: MycoKeys. 2019 Dec 11;61:39–74. doi: 10.3897/mycokeys.61.38175 (PMC6920222; doi:10.3897/mycokeys.61.38175)
Supplement: Supplementary material 1 [file mycokeys-61-039-s004.pdf]

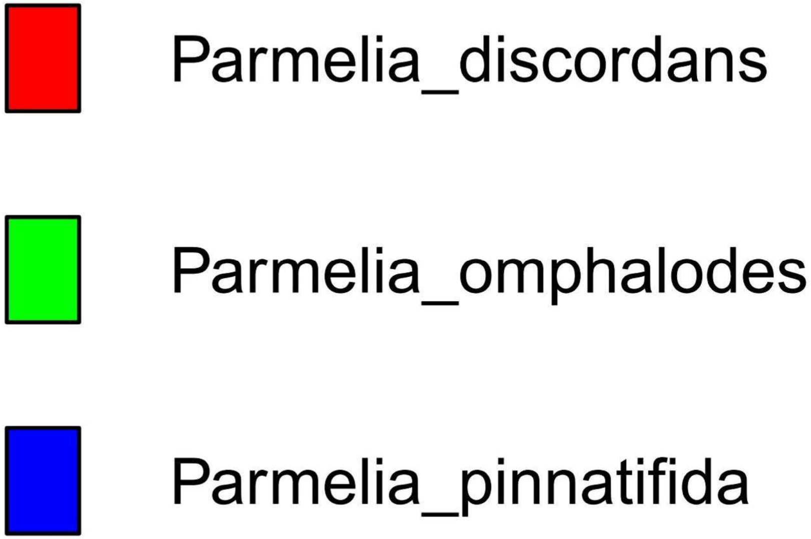
Parmelia_discordans
Parmelia_omphalodes
Parmelia_pinnatifida

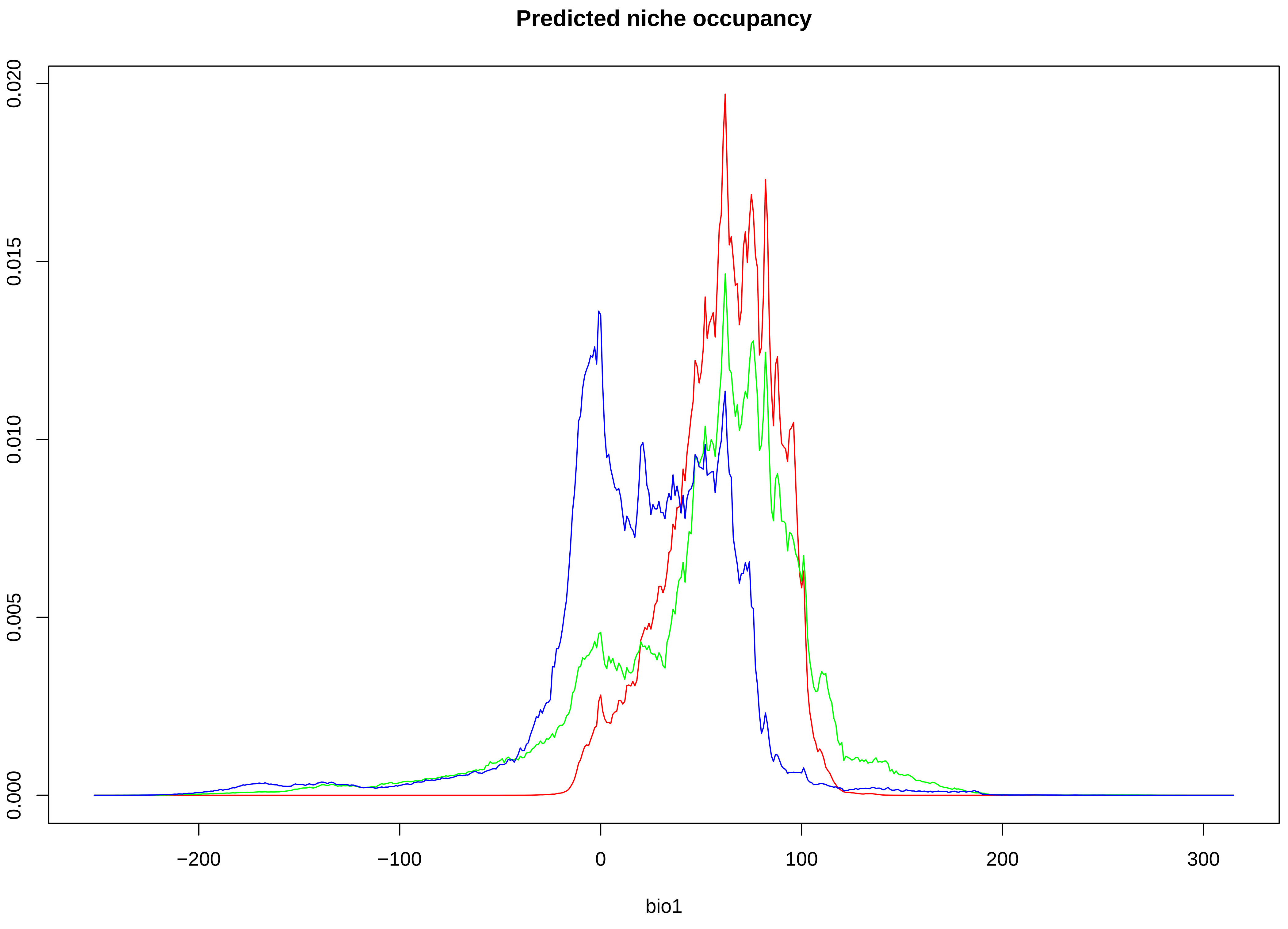

Predicted niche occupancy
0.000
0.005
0.010
0.015
0.020
−200
−100
0
100
200
300
bio1

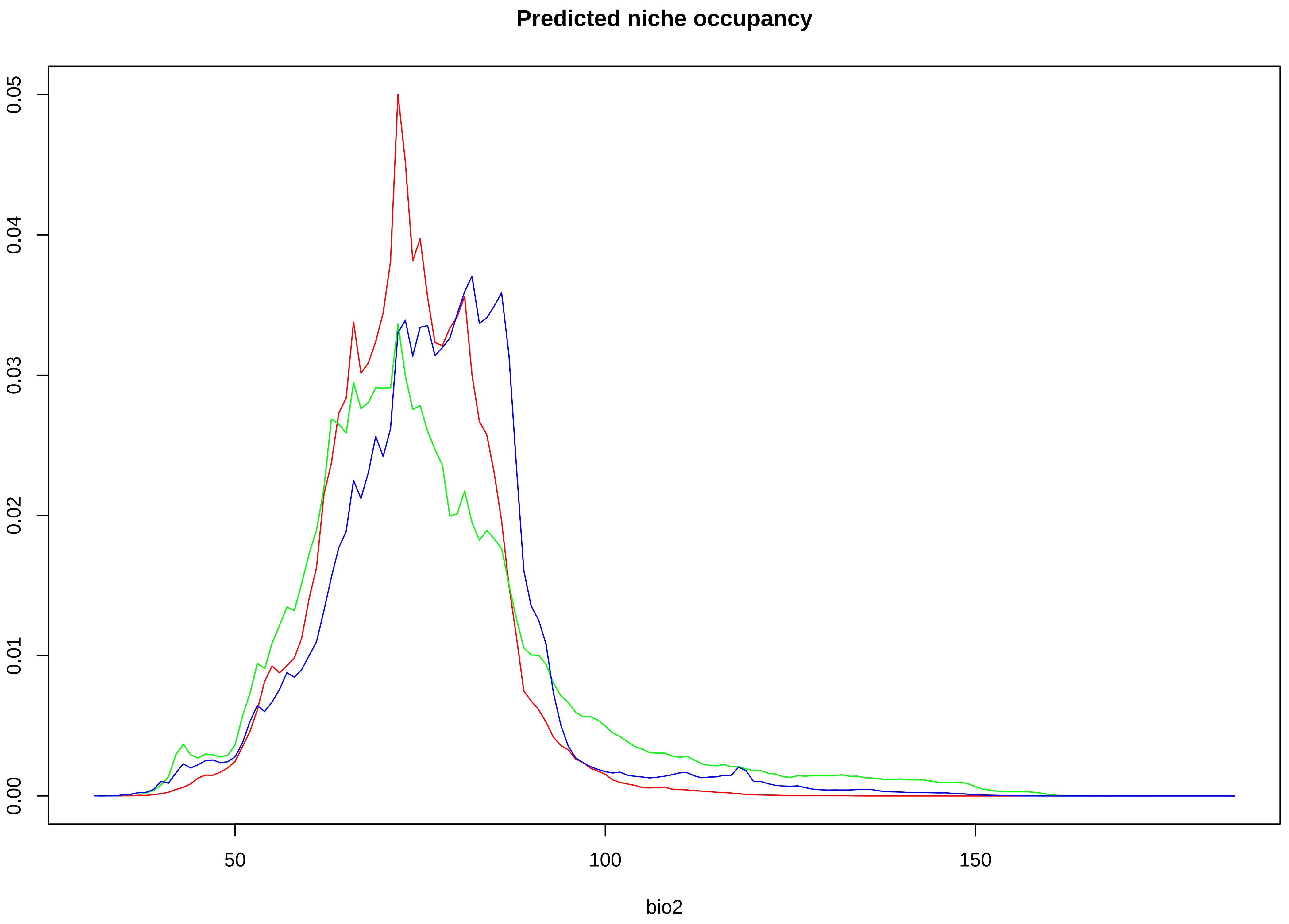
Predicted niche occupancy
0.00
0.01
0.02
0.03
0.04
0.05
50
100
150
bio2

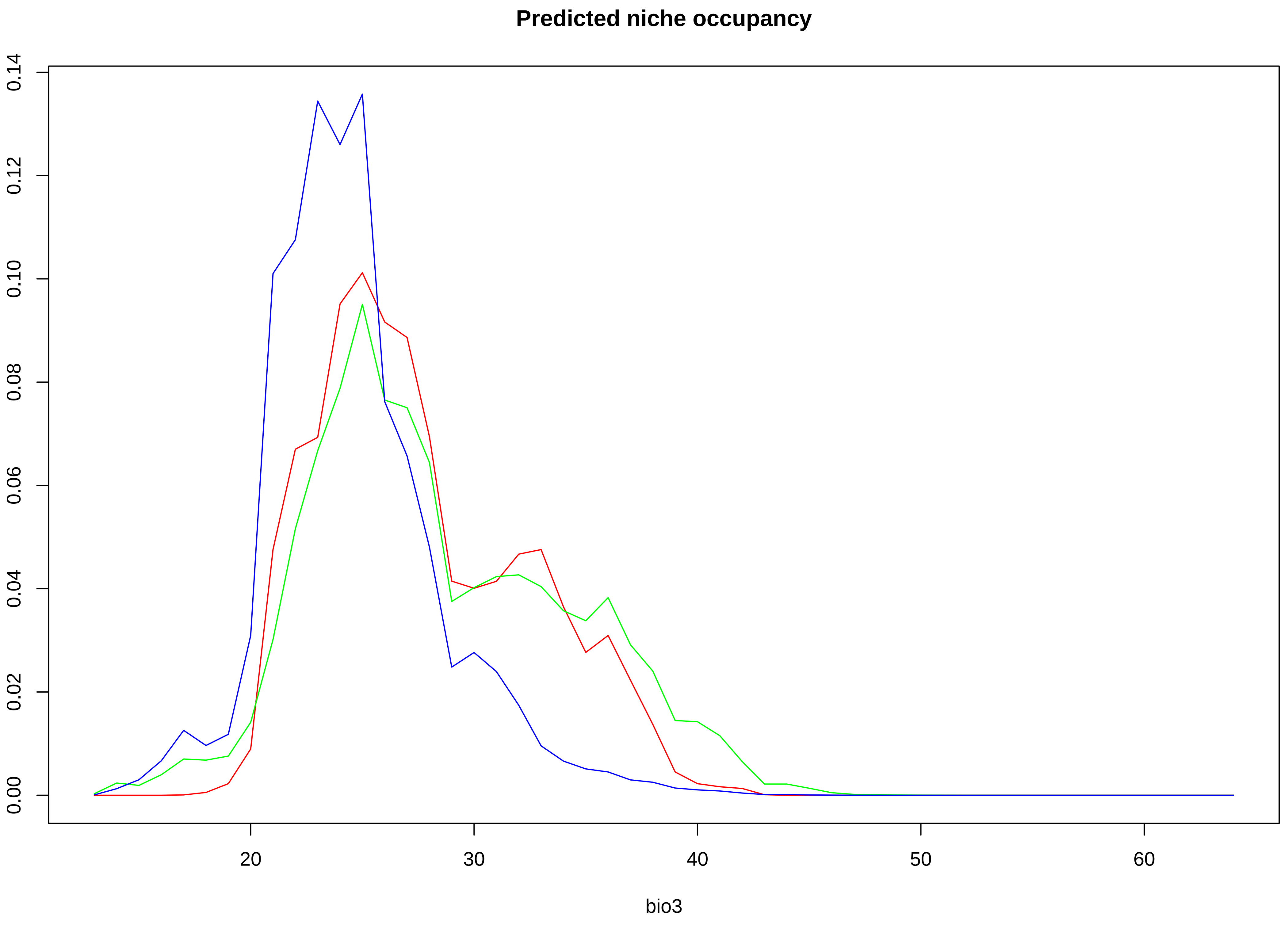
Predicted niche occupancy
0.00
0.02
0.04
0.06
0.08
0.10
0.12
0.14
20
30
40
50
60
bio3

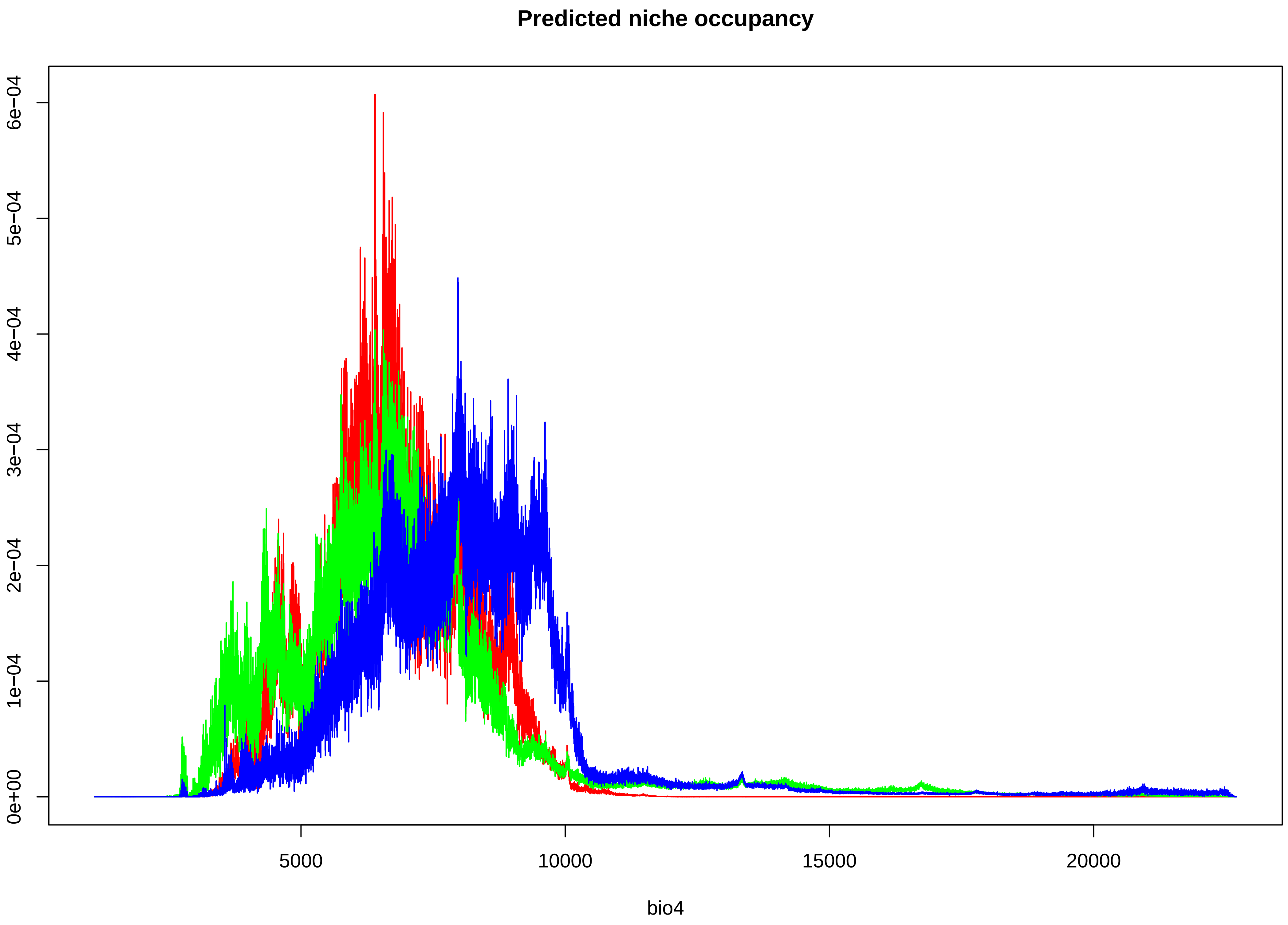
Predicted niche occupancy
0e+00
1e−04
2e−04
3e−04
4e−04
5e−04
6e−04
5000
10000
15000
20000
bio4

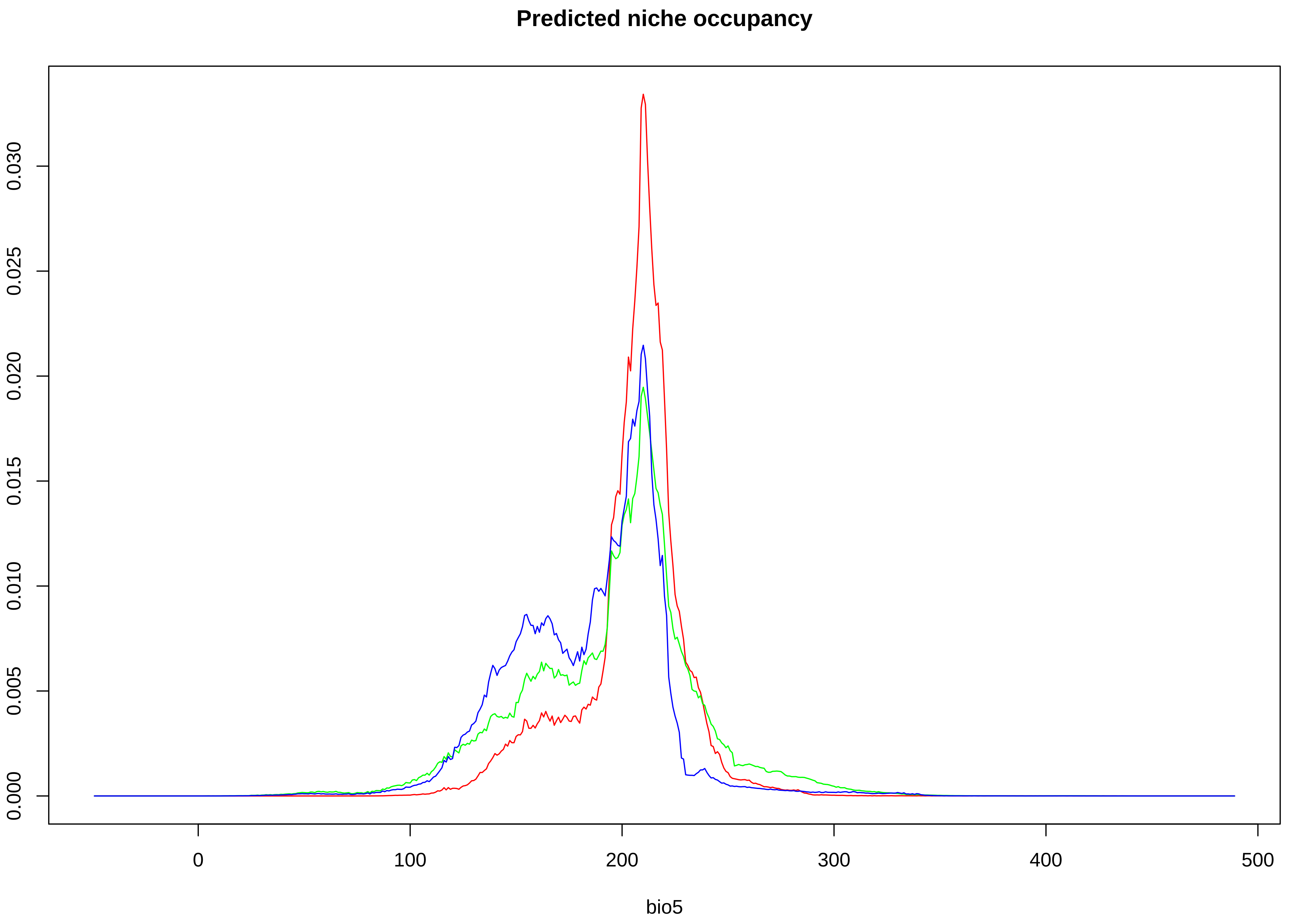

Predicted niche occupancy
0.000
0.005
0.010
0.015
0.020
0.025
0.030
0
100
200
300
400
500
bio5

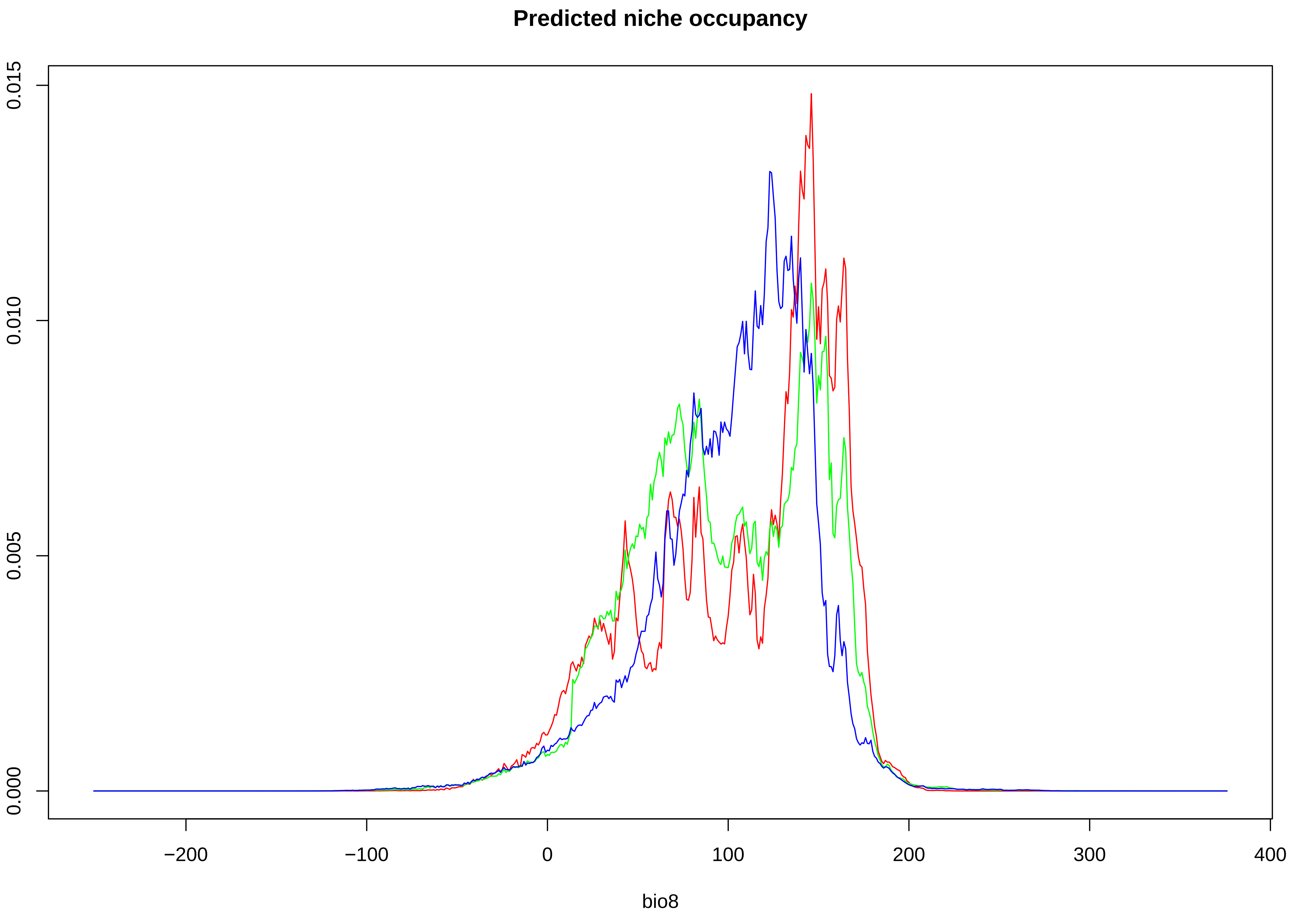
Predicted niche occupancy
0.000
0.005
0.010
0.015
−200
−100
0
100
200
300
400
bio8

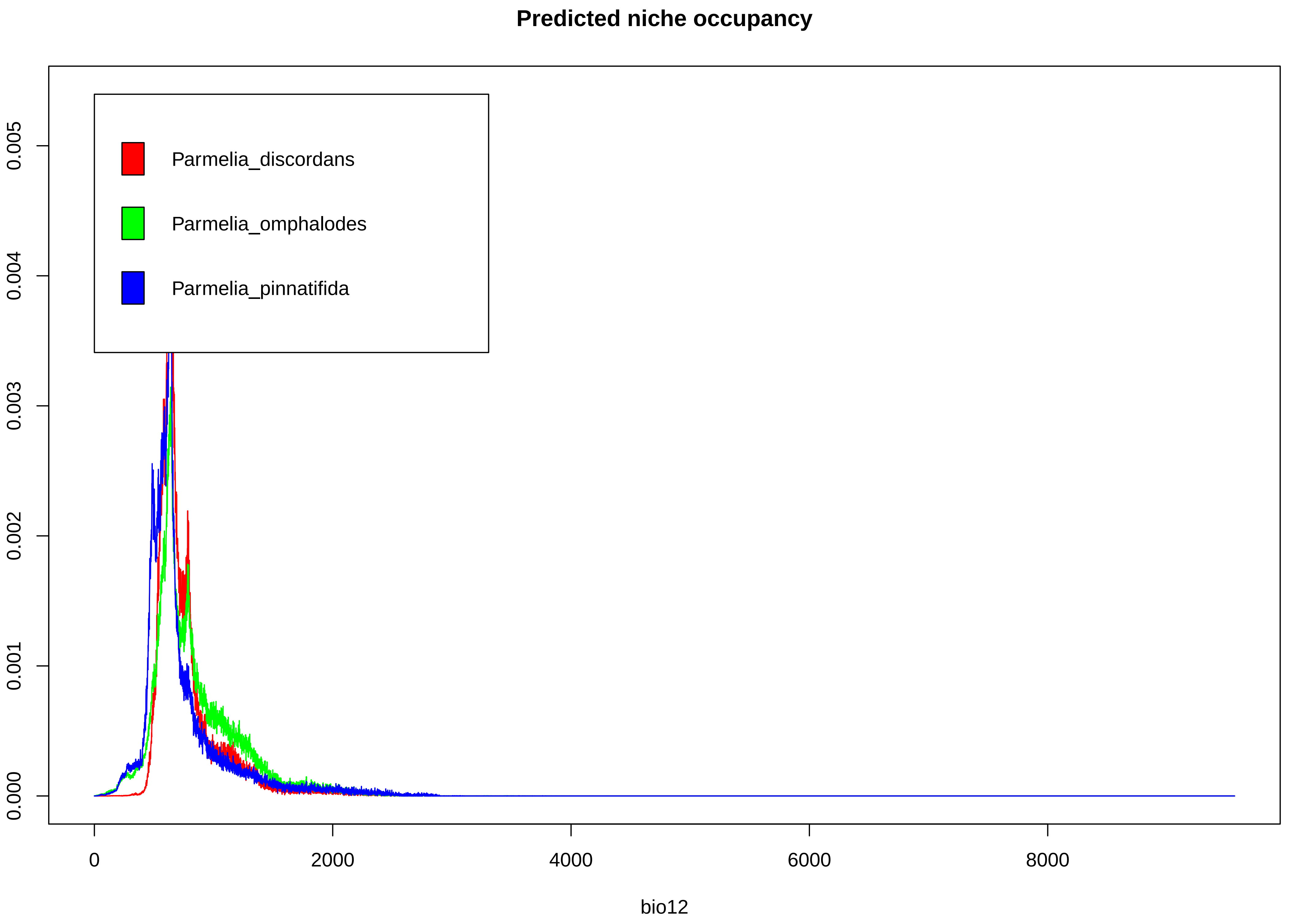

Predicted niche occupancy
Parmelia_discordans
Parmelia_omphalodes
Parmelia_pinnatifida
0.000
0.001
0.002
0.003
0.004
0.005
0
2000
4000
6000
8000
bio12

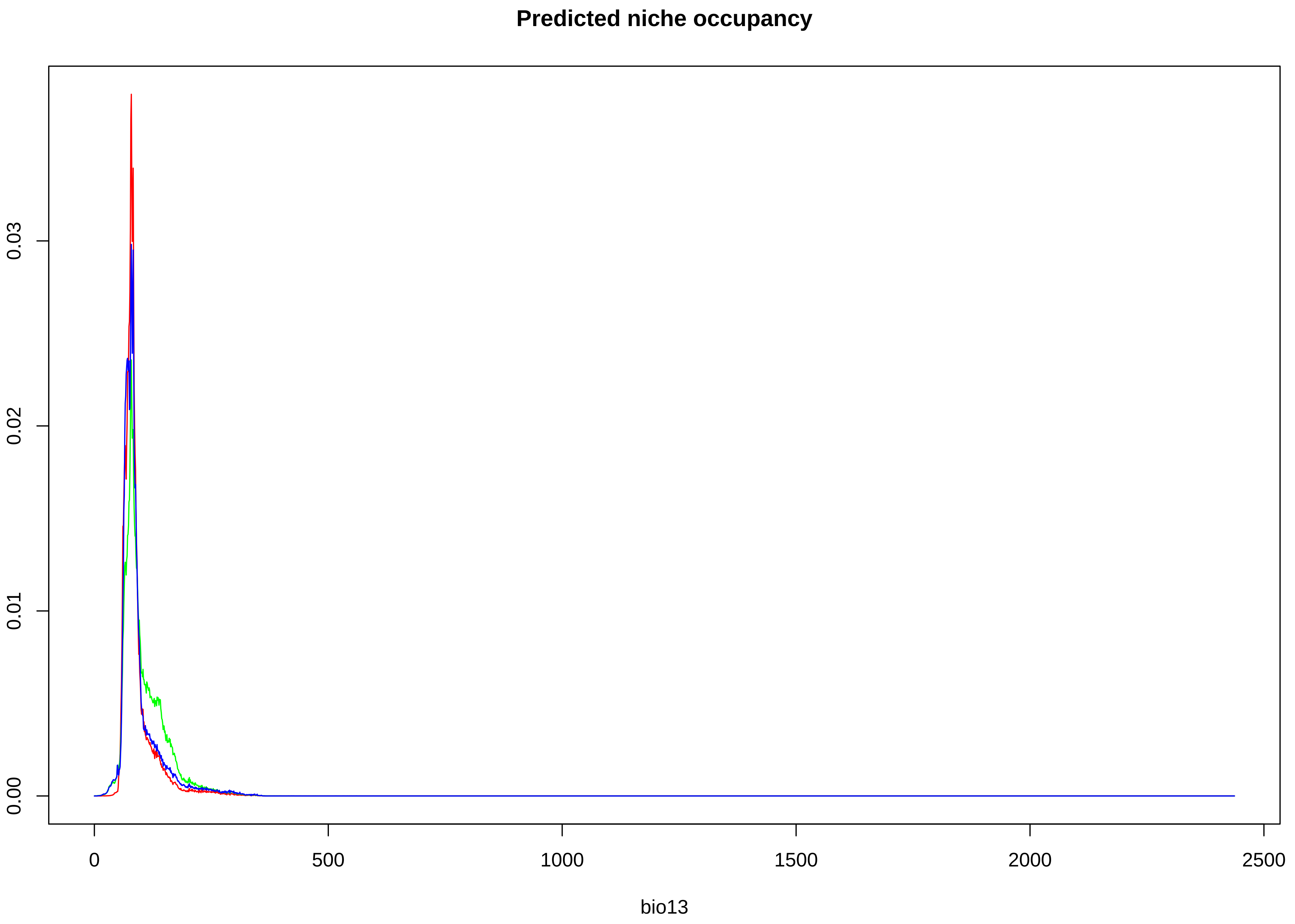
Predicted niche occupancy
0.00
0.01
0.02
0.03
0
500
1000
1500
2000
2500
bio13

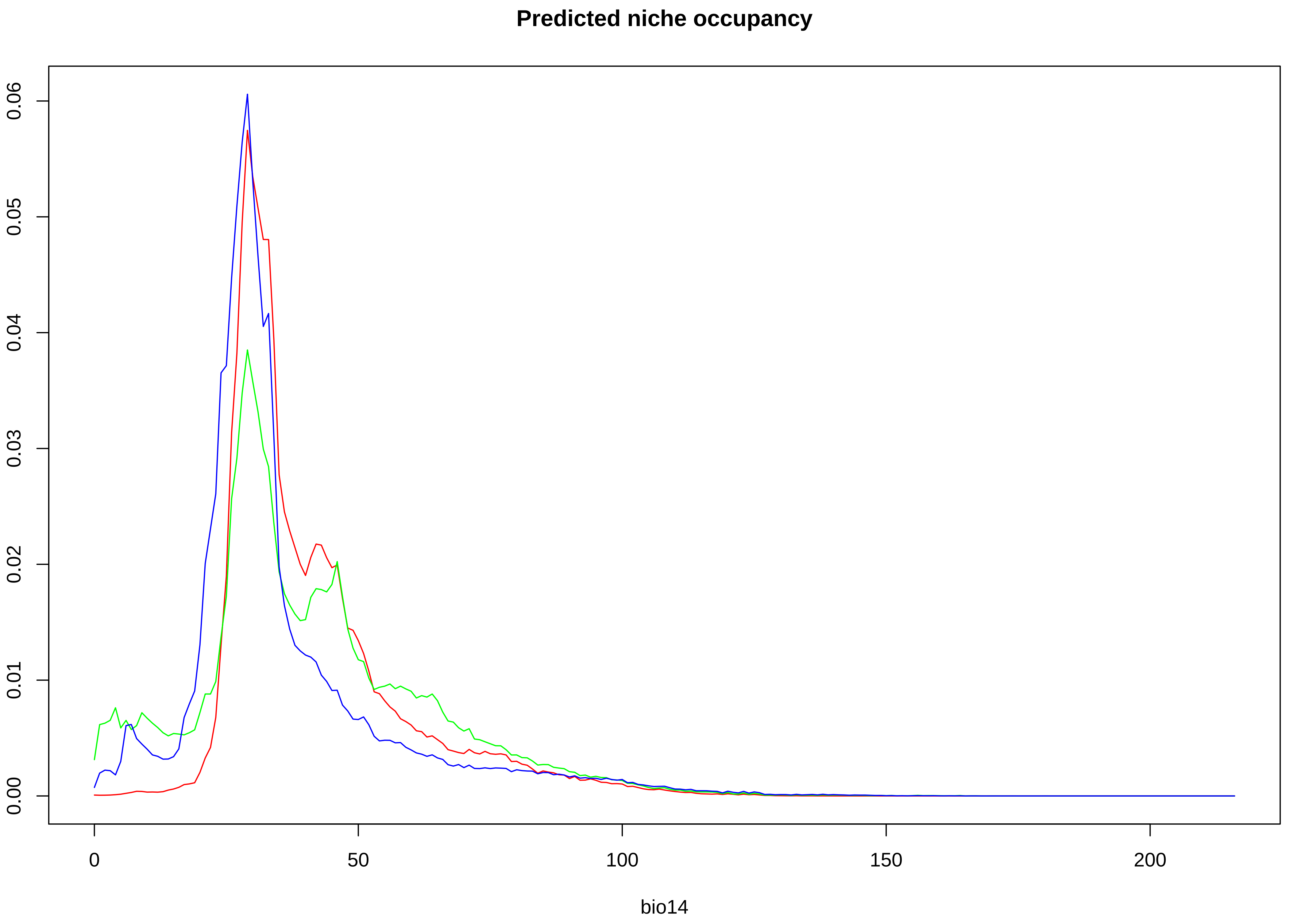
Predicted niche occupancy
0.00
0.01
0.02
0.03
0.04
0.05
0.06
0
50
100
150
200
bio14

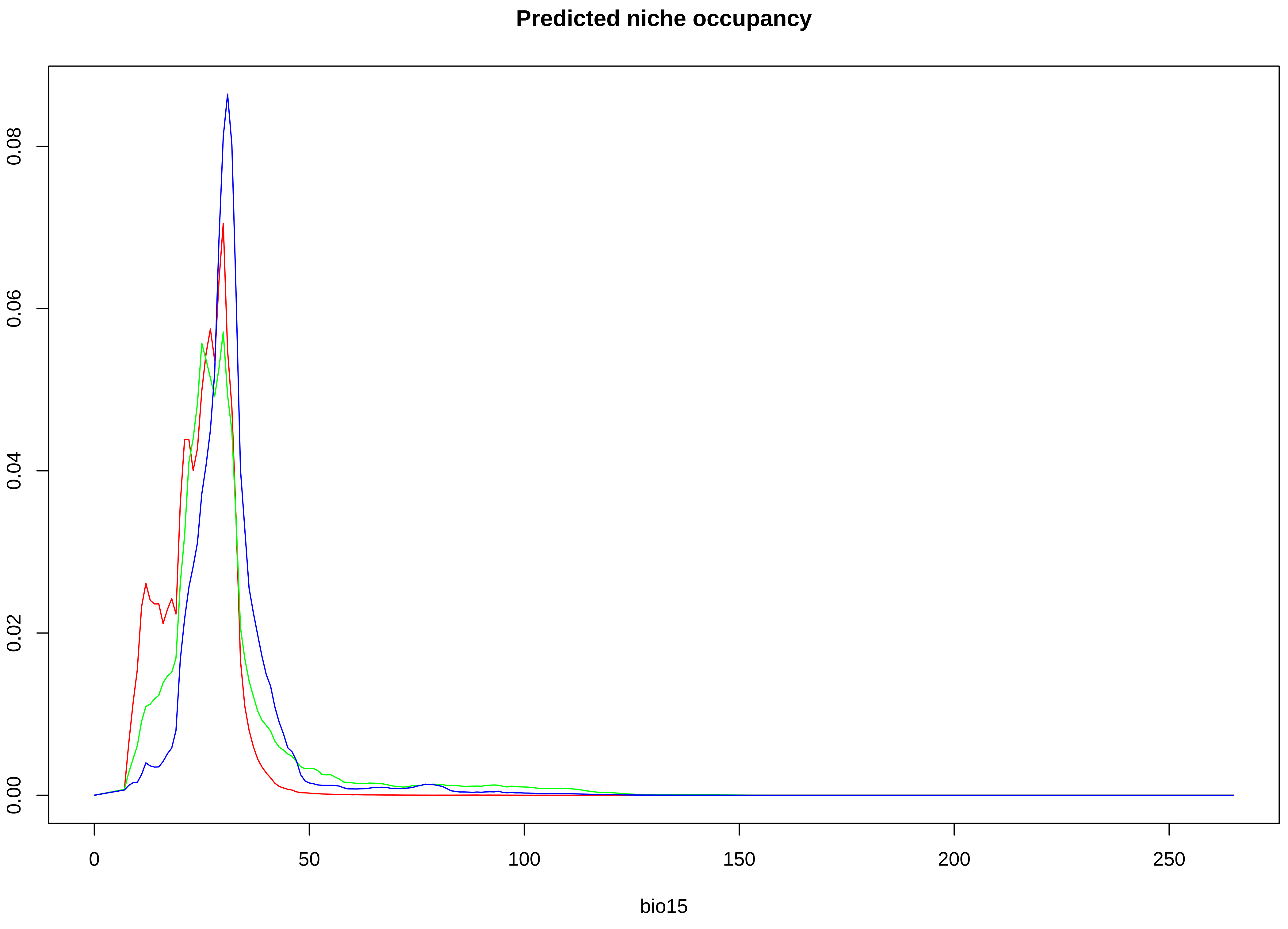
Predicted niche occupancy
0.00
0.02
0.04
0.06
0.08
0
50
100
150
200
250
bio15

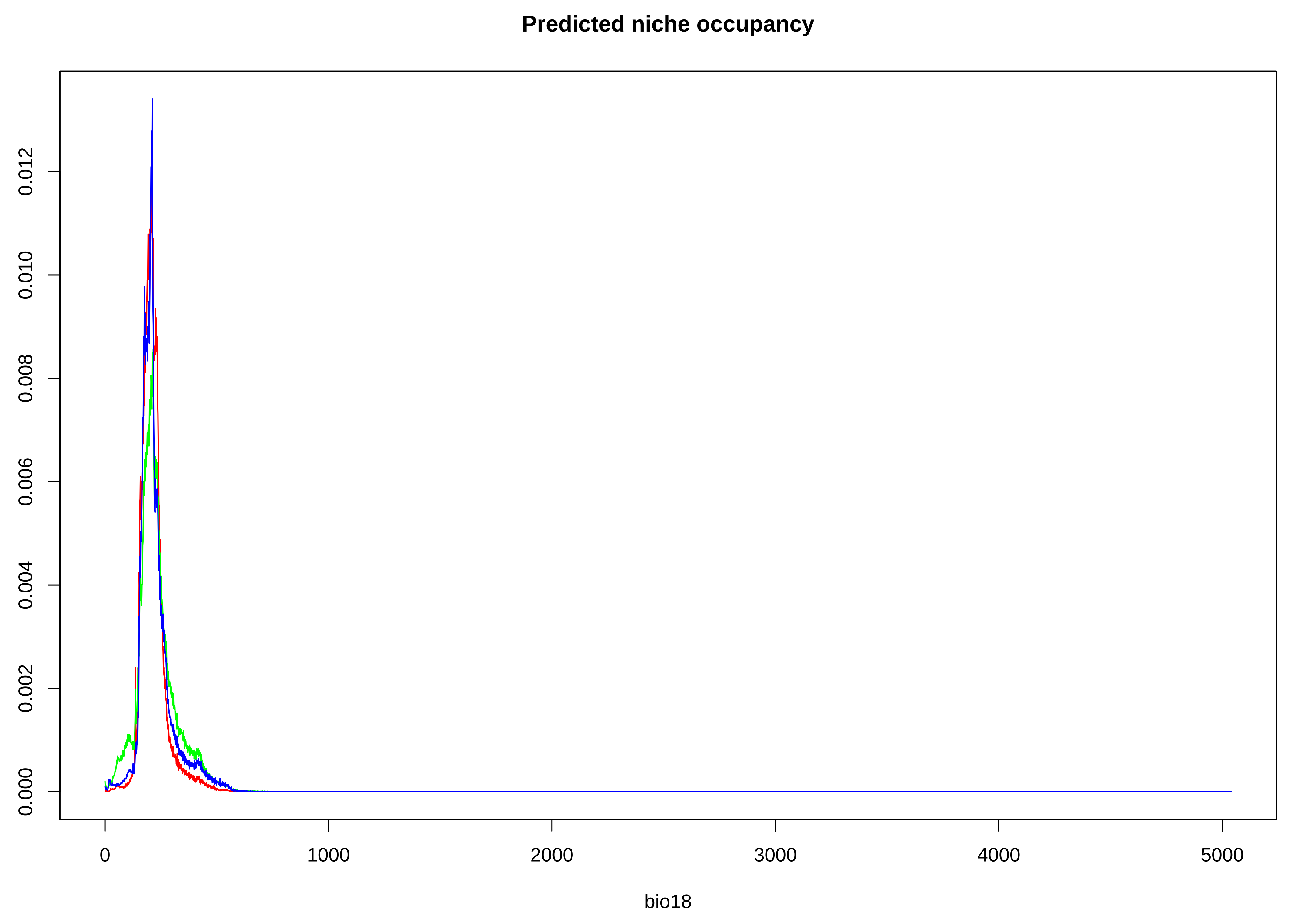

Predicted niche occupancy
0.000
0.002
0.004
0.006
0.008
0.010
0.012
0
1000
2000
3000
4000
5000
bio18

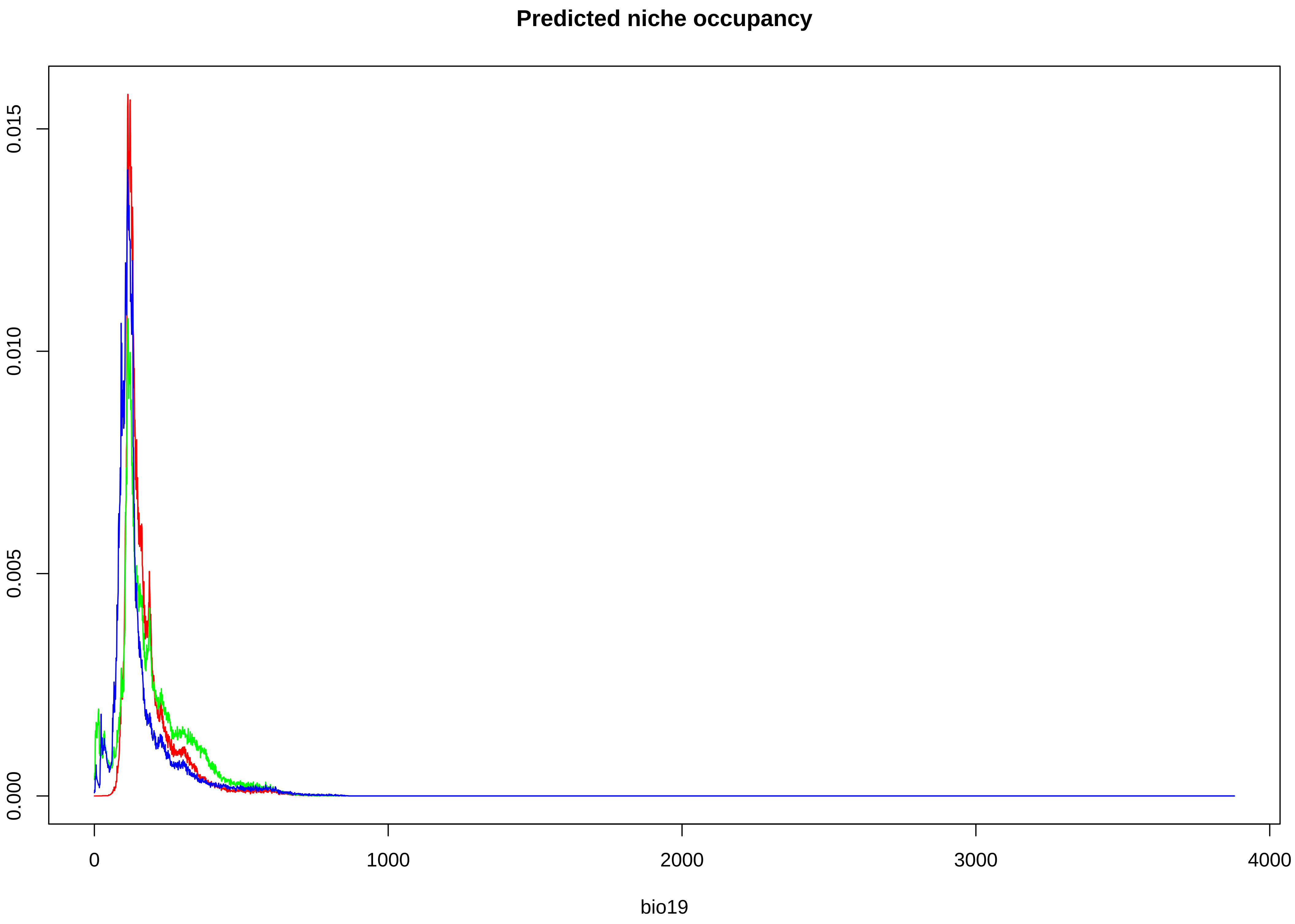

Predicted niche occupancy
0.000
0.005
0.010
0.015
0
1000
2000
3000
4000
bio19
